# Prevention of cardiac surgery-associated acute kidney injury: a systematic review and meta-analysis of non-pharmacological interventions

**DOI:** 10.1186/s13054-023-04640-1

**Published:** 2023-09-12

**Authors:** Geoffroy Hariri, Lucie Collet, Lucie Duarte, Guillaume L. Martin, Matthieu Resche-Rigon, Guillaume Lebreton, Adrien Bouglé, Agnès Dechartres

**Affiliations:** 1 Département de Santé Publique, UMR-S 1136, AP-HP, INSERM, Institut Pierre Louis d’Epidémiologie et de Santé Publique, Hôpital Pitié-Salpêtrière, Sorbonne Université, Paris, France; 2 Département d’anesthésie et réanimation, GRC 29, DMU DREAM, Institut de Cardiologie, Assistance Publique-Hôpitaux de Paris (AP-HP), Hôpital La Pitié-Salpêtrière, Sorbonne Université, 75013 Paris, France; 3 ECSTRRA - CRESS UMR1153, INSERM and SBIM, AP-HP, Hôpital Saint-Louis, Université de Paris, Paris, France; 4 AP-HP, Service de Chirurgie Cardiaque, Institut de Cardiologie, Hôpital La Pitié-Salpêtrière, Sorbonne Université, 75013 Paris, France

**Keywords:** Cardiac surgery, Acute kidney injury, Non-pharmacological interventions, Prevention

## Abstract

**Background:**

Cardiac surgery-associated acute kidney injury (CSA-AKI) is frequent. While two network meta-analyses assessed the impact of pharmacological interventions to prevent CSA-AKI, none focused on non-pharmacological interventions. We aim to assess the effectiveness of non-pharmacological interventions to reduce the incidence of CSA-AKI.

**Methods:**

We searched PubMed, Embase, Central and clinical trial registries from January 1, 2004 (first consensus definition of AKI) to July 1, 2023. Additionally, we conducted manual screening of abstracts of major anesthesia and intensive care conferences over the last 5 years and reference lists of relevant studies. We selected all randomized controlled trials (RCTs) assessing a non-pharmacological intervention to reduce the incidence of CSA-AKI, without language restriction. We excluded RCTs of heart transplantation or involving a pediatric population. The primary outcome variable was CSA-AKI. Two reviewers independently identified trials, extracted data and assessed risk of bias. Random-effects meta-analyses were conducted to calculate risk ratios (RRs) with 95% confidence intervals (CIs). We used the Grading of Recommendations Assessment, Development, and Evaluation to assess the quality of evidence.

**Results:**

We included 86 trials (25,855 patients) evaluating 10 non-pharmacological interventions to reduce the incidence of CSA-AKI. No intervention had high-quality evidence to reduce CSA-AKI. Two interventions were associated with a significant reduction in CSA-AKI incidence, with moderate quality of evidence: goal-directed perfusion (RR, 0.55 [95% CI 0.40–0.76], *I*^2^ = 0%; *P*_het_ = 0.44) and remote ischemic preconditioning (RR, 0.86 [0.78–0.95]; *I*^2^ = 23%; *P*_het_ = 0.07). Pulsatile flow during cardiopulmonary bypass was associated with a significant reduction in CSA-AKI incidence but with very low quality of evidence (RR = 0.69 [0.48; 0.99]; *I*^2^ = 53%; *P*_het_ < 0.01). We found high quality of evidence for lack of effect of restrictive transfusion strategy (RR, 1.02 [95% CI 0.92; 1.12; *P*_het_ = 0.67; *I*^2^ = 3%) and tight glycemic control (RR, 0.86 [95% CI 0.55; 1.35]; *P*_het_ = 0.25; *I*^2^ = 26%).

**Conclusions:**

Two non-pharmacological interventions are likely to reduce CSA-AKI incidence, with moderate quality of evidence: goal-directed perfusion and remote ischemic preconditioning.

**Supplementary Information:**

The online version contains supplementary material available at 10.1186/s13054-023-04640-1.

## Introduction

The development of acute kidney injury (AKI) after cardiac surgery, known as cardiac surgery-associated acute kidney injury (CSA-AKI), is frequent. The incidence is estimated at 20–40% of patients undergoing cardiac surgery, with renal replacement therapy (RRT) required in 1.6–5.8% of cases [1–3]. The occurrence of AKI is a major complication worsening the prognosis after cardiac surgery, increasing peri-operative mortality by a factor of 3–8 [4–6]. In the long term, CSA-AKI is independently associated with increased risk of end-stage renal disease and death [7, 8].

Many mechanisms are involved in the development of CSA-AKI. Cardiac surgery is frequently associated with low systemic output secondary to cardiopulmonary bypass (CPB) [9, 10] leading to renal ischemia [11]. In addition, ischemia–reperfusion injury after CPB may worsen renal injury because of oxidative stress and inflammatory response. Finally, hemolysis during CPB leads to nitric oxide scavenging, thus resulting in vasoconstriction and reduced renal perfusion [12]

Many pharmacological and non-pharmacological interventions have been tested to reducing the incidence of CSA-AKI. However, randomized controlled trials (RCTs) have presented discordant results [13]. Several meta-analyses were carried out, but none clearly demonstrated the superiority of a particular intervention to reduce the incidence of CSA-AKI [14–16]. Recently, 2 network meta-analyses found that natriuretic peptide may be the best pharmacological intervention to prevent CSA-AKI [17, 18]. However, these studies did not consider non-pharmacological interventions, which could be of interest in preventing CSA-AKI.

In this systematic review and meta-analysis, we assessed the effectiveness of non-pharmacological interventions to reduce the incidence of CSA-AKI.

## Methods

This article follows the PRISMA statement [19]. The protocol was registered on PROSPERO (CRD42021255779) with changes reported in Additional file 1: Table S1.

### Eligibility criteria

We included only RCTs. There was no restriction on publication status or language, but we selected trials published after 2004, the year of publication of the RIFLE criteria for AKI [20]. We considered adults undergoing cardiac surgery, such as coronary artery bypass grafting (CABG) and/or valve surgery. We did not include trials of heart transplantation because of the specificity of certain interventions and percutaneous cardiac interventions because of their low risk of renal injury. We considered all non-pharmacological interventions except biocompatible coating because of the large number of different coated circuits. All comparators were eligible. Our primary outcome was CSA-AKI. Secondary outcomes were hospital mortality, post-operative new-onset dialysis, length of ICU and hospital stay, post-operative myocardial infarction, atrial fibrillation and stroke.

### Search strategy

We searched PubMed, Embase and Central from January 1, 2004 to July 1, 2021, updated to July 1, 2023, by using a dedicated search algorithm (Additional file 1). We also searched ClinicalTrials.gov, the European Union Clinical Trials Registry and the WHO International Clinical Trials Registry Platform and manually screened the abstracts of the major anesthesia and intensive care conferences over the last 5 years (American Society of Anesthesiology, European Society of Anesthesiology, European Society of Intensive Care Medicine, Société Française d’Anesthésie et Réanimation, Société de Reanimation de Langue Française). Finally, we screened the reference lists of the selected studies and previous systematic reviews/meta-analyses.

### Selection process

Two reviewers (GH and LC/LD) independently screened the title and abstract of all references, then the full text. Any discrepancies were resolved with a third reviewer (AD) to reach consensus. If an article contained insufficient data, we contacted the corresponding author.

### Data extraction

For each trial, 2 reviewers (GH and LC/LD) independently extracted the following data by using a standardized data extraction form.

- General characteristics: first author, year and journal of publication, language, countries, funding source, design, recruitment period, number of study centers.
- Patient characteristics: main eligibility criteria, age, sex, type of cardiac surgery.
- Intervention characteristics: type and timing (pre-, intra-, post-operative).
- Control group: other intervention, its type and timing.
- Definition of CSA-AKI: consensus classification (RIFLE, Acute Kidney Injury Network [AKIN], Kidney Disease Improving Global Outcomes [KDIGO]), other definitions (increased creatinine level, RRT) or not reported.
- Results: for each group, the number of events and number of patients analyzed for binary outcomes and mean, standard deviation and number of patients analyzed for continuous outcomes. When necessary, we converted results from median and range and/or interquartile range to mean and standard deviation [21].

Corresponding authors were contacted in case of missing data.

### Risk of bias assessment

Two reviewers (GH and LC) independently assessed the risk of bias for each trial with the revised version of the Cochrane Risk of Bias tool (RoB 2.0) for RCTs [22].

### Data synthesis and analysis

We originally planned a network meta-analysis, but all RCTs compared the experimental intervention to usual care and there were no comparisons between different interventions. Therefore, we performed conventional meta-analyses for each intervention using random-effects models. We estimated risk ratios (RRs) with 95% confidence intervals (95% CIs) for binary outcomes and mean differences (MD) with 95% CIs for continuous outcomes. We assessed the heterogeneity across trials by visually inspecting forest plots and by the Cochran *Q* test and *I*^2^ [23].

When enough trials were available, we performed subgroup analyses for CSA-AKI according to the type of surgery (CABG, valve surgery or combined), the definition of CSA-AKI (RIFLE, AKIN, KDIGO, increased creatinine level, RRT or not reported), the pre-operative renal status (chronic kidney disease or not) and left ventricular function (poor or not), the timing of the intervention (pre-, intra- or postoperative) and overall risk of bias of the trial (low, some concerns, high). For pulsatile blood flow during CPB, we performed a post-hoc subgroup analysis to assess the effect of the 2 main modalities (intra-aortic balloon pump [IABP] during CPB or pulsatile CPB).

We used funnel plots to evaluate the presence of small-study effects in each meta-analysis including 10 trials or more with the Egger test [24].

All statistical analyses were conducted with R 4.2.1 [25]. A two-sided *p* < 0.05 was considered statistically significant.

### Grading the quality of evidence

For each intervention, we rated the quality of evidence for CSA-AKI according to the Grading of Recommendation, Assessment, Development, and Evaluation (GRADE) Working Group system [26].

## Results

### Description of included trials

**Fig. 1:**
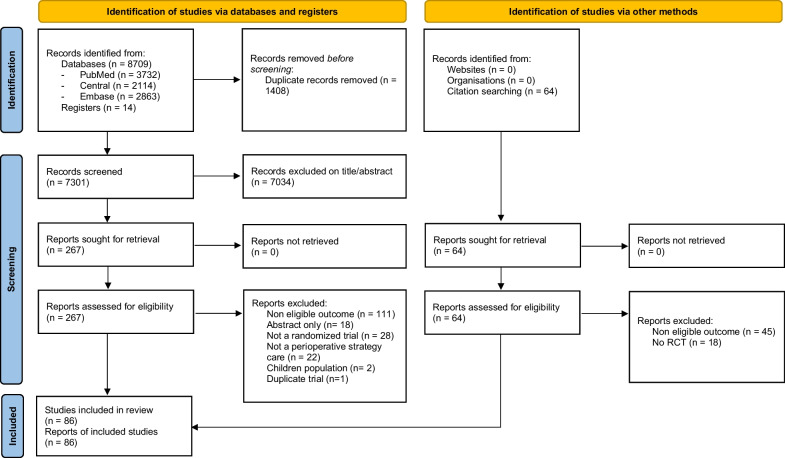
Flow diagram of study selection

From 7301 unique citations, 267 trials were eligible for full-text analysis. Finally, 86 RCTs including 25,855 patients were selected (Fig. 1). These trials evaluated 10 non-pharmacological interventions, the most frequent being remote ischemic preconditioning (RIPc) (31 RCTs, 7738 patients), minimally invasive extracorporeal circuit (MECC) (14 RCTs, 1617 patients) and pulsatile blood flow during CPB (10 RCTs, 1993 patients). The median year of publication was 2015 (IQR, 2011-2018). The median sample size was 114 participants (IQR, 78–245). Patient mean age was 65 years (SD, 9). Nearly half of the trials focused on CABG (*n* = 36 [42%]). Most trials included patients with an on-pump procedure (*n* = 74 [86%]) (Table 1 and Additional file 1: Table S2). Only 3 (3%) trials focused on patients with chronic kidney disease (CKD) for inclusion criteria while 41(39%) excluded patients with advanced CKD (Additional file 1: Tables S2 and S3).

**Table 1:** General characteristics of included trials by non-pharmacological intervention evaluated

Characteristics	Total	GDP	RIPc	Pulsatile flow during CPB	MECC	Restrictive transfusion strategy	Epidural analgesia	Tight glycemic control	KDIGO care	High- Target arterial pressure	Hyperoxia during CPB
*Number of RCTs, n*	86	2	31	10	14	6	4	10	3	3	3
*Number of participants, n*	25 855	601	7738	1993	1617	8289	903	2753	662	577	722
Median (IQR)	114 (78–245)	300 (287–313)	100 (71–190)	134 (89–219)	85 (68–118)	609 (243––680)	94 (75–244)	122 (96–197)	276 (192–277)	195 (142–243)	298 (199–311)
Event rates, *n* (%)											
Intervention	2422 (19)	44 (15)	855 (22)	121 (12)	46 (6)	956 (23)	18 (4)	53 (4)	142 (44)	53 (18)	134 (37)
Control	2660 ((20)	84 (27)	932 (24)	194 (19)	67 (8)	944 (22)	20 (4)	76 (5)	162 (48)	42 (15)	139 (38)
Publication year, median (IQR)	2015 (2011–2018)	2019 (2018–2020)	2015 (2013–2017)	2011 (2009–2012)	2014 (2008–2016)	2016 (2013–2017)	2012 (2009–2013)	2014 (2010–2019)	2017 (2017–2019)	2018 (2016–2018)	2019 (2017–2020)
*Location, n (%)*											
Europe	43 (50)	–	12 (38)	8 (72)	11 (78)	2 (33)	3 (75)	–	3 (100)	3 (100)	1 (33)
North America	7 (8)	–	2 (6)	–	–	2 (33)	–	3 (30)	–	–	–
Asia	26 (30)	1 (50)	15 (48)	2 (18)	3 (21)	–	1 (25)	3 (30)	–	–	1 (33)
Other	10(11)	1 (50)	2 (6)	–	–	2 (33)	–	4 (40)	–	–	1 (33)
*Study design, n (%)*											
Superiority	53 (61)	2 (100)	24 (77)	5 (45)	5 (35)	3 (50)	2 (50)	5 (50)	2 (67)	2 (67)	3 (100)
Non-inferiority	4 (5)	–	–	–	–	2 (40)	–	1 (10)	1 (33)	–	–
*Recruitment, n (%)*											
Single center	69 (80)	1 (50)	26 (84)	10 (100)	14 (100)	2 (33)	3 (75)	8 (80)	2 (67)	3 (100)	–
Multicenter	17 (20)	1 (50)	5 (16)		–	4 (67)	1 (25)	2 (20)	1 (33)	–	3 (100)
*Funding source, n (%)*											
Public	30 (35)	–	16 (51)	–	3 (21)	2 (33)	–	3 (30)	1 (33)	3 (100)	2 (66)
Private	4 (5)	–	1 (3)	–	1 (7)	1 (16)	–	1 (10)	–	–	–
Mixed	7 (8)	1 (50)	2 (6)	–	1 (7)	1 (16)	–	1 (10)	1 (33)	–	–
*Cardiopulmonary bypass, n (%)*											
On-pump	74 (86)	2 (100)	25 (80)	10 (100)	14 (100)	5 (84)	1 (25)	8 (80)	3 (100)	3 (100)	3 (100)
Off-Pump	5 (6)	–	4 (12)	_	–		1 (25)		–	–	–
Both	7 (8)	–	2 (8)	_	–	1 (16)	2 (50)	2 (20)	–	–	–
*Surgical procedure, n (%)*											
CABG	36 (42)	–	10 (33)	8 (72)	11 (78)	–	1 (25)	5 (50)	–	–	1 (33)
Valve replacement	8 (9)	–	6 (20)	–	2 (14)	–	–	–	–	–	–
CABG or valve replacement	4 (5)	–	2 (6)	2 (18)	–	–	–	–	–	–	–
Valve replacement or combined	2 (2)	–	1 (3)	–	1 (7)		–	–		–	–
Combined	2 (2)	–	1 (3)	–	–	–	–	–	–	1 (33)	–
All	33 (38)	2 (100)	10 (30)	–	–	6 (100)	3 (75)	5 (50)	3 (100)	2 (67)	2 (67)
Aortic arch replacement	1 (1)	–	1 (3)		–			–	–	–	–
*AKI definition, n (%)*											
KDIGO	17 (20)	1 (50)	7 (22)	–	–	2 (33)	–	1 (10)	3 (100)	–	3 (100)
AKIN	23 (27)	1 (50)	14 (45)	1 (9)	5 (35)	1 (16)	1 (25)	–	–	–	–
RIFLE	6 (7)	–	3 (9)	–	1 (7)	1 (16)	–	–	–	1 (33)	–
Creatinine level increased	25 (29)	–	4 (13)	6 (64)	5 (35)	1 (16)	3 (75)	4 (40)	–	2 (67)	–
RRT	3 (4)	–	–	–	1 (7)	–	–	2 (20)	–	–	–
Not reported	12 (14)	–	3 (9)	3 (27)	2 (14)	1 (16)	–	3 (30)	–	–	–

*RCT*, randomized controlled trial; *GDP*: goal-directed perfusion; *RIPc*, remote ischemic preconditioning; *MECC*, minimally invasive extracorporeal circulation; *IQR*, interquartile range; *RRT*, renal replacement therapy; *CABG*, coronary artery bypass grafting; *CPB*, cardiopulmonary bypass; *KDIGO*, kidney disease improving global outcomes; *AKIN*, acute kidney injury network; *RIFLE*, risk [class R], injury [class I], failure [class F], loss [class L] and end stage renal disease [class E])

The overall risk of bias was assessed as high for 26 trials (30%), some concerns for 13 (15%) and low for 47 (55%) (Additional file 1: Figure S1). The main domain at high risk of bias was the selection of reporting results for 19 trials (22%).

### Incidence and definition of AKI

Overall, 5082 (20%) patients developed CSA-AKI. Half of the trials (*n* = 46) had a definition of AKI using a consensus classification: AKIN (*n* = 23 [27%]), KDIGO (*n* = 17 [20%]) or RIFLE (*n* = 6 [7%]). Among trials using consensus classification of AKI, only 25 (54%) were based on both creatinine and urine output assessment. Severity of AKI was reported in 28 (61%) trials including 7842 patients. Among them, 2283 (29%) developed AKI: 64% of patients (*n* = 1467) had mild AKI (stage 1 or Risk), 23% (*n* = 520) had moderate AKI (stage 2 or injury) and 13% (*n* = 293) had severe AKI (stage 3 or failure). The other definitions of AKI included elevated creatinine level (*n* = 25 [29%]) or the use of RRT (*n* = 3 [4%]). Twelve trials (14%) did not state how CSA-AKI was defined (Additional file 1: Figure S2a). The proportion of trials using a consensus classification of AKI increased over time (Additional file 1: Figure S2b).

### Principal findings

**Fig. 2:**
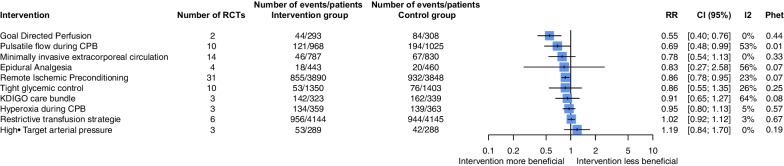
Forest plot of non-pharmacological interventions for preventing cardiac surgery-associated acute kidney injury. Interventions were compared to standard medical care as a control. CPB, cardiopulmonary bypass; KDIGO, Kidney Disease Improving Global Outcomes; RCTs, randomized controlled trials

Three interventions were associated with a significantly reduced risk of CSA-AKI: goal-directed perfusion (GDP), RIPc and pulsatile flow during CPB (Fig. 2).

#### Interventions associated with significant reduction in CSA-AKI incidence

##### GDP

We included 2 trials (601 patients), both at low risk of bias. The definition of AKI was KDIGO for one trial and AKIN for the other. In both trials, the GDP strategy was to maintain oxygen delivery (DO2) above a minimal target (280 or 300 mL/min/m2) during CPB.

The GDP strategy was associated with significantly reduced incidence of CSA-AKI as compared with usual care (RR, 0.55 [95% CI 0.40–0.76]). Heterogeneity was low (*I*^2^ = 0%; *P*_het_ = 0.44) (Fig. 2; Additional file 1: Figure S3).

No subgroup analysis was possible for this intervention.

#### RIPc

RIPc was evaluated in 31 RCTs (7738 patients). Most trials were at low risk of bias (*n* = 20 [65%]), 6 (19%) high risk of bias and 5 (16%) some concerns. The definition of AKI was a consensus classification in 24 trials (77%) (AKIN: 14 [45%]; KDIGO: 7 [22%]; RIFLE: 3 [9%]).

**Fig. 3:**
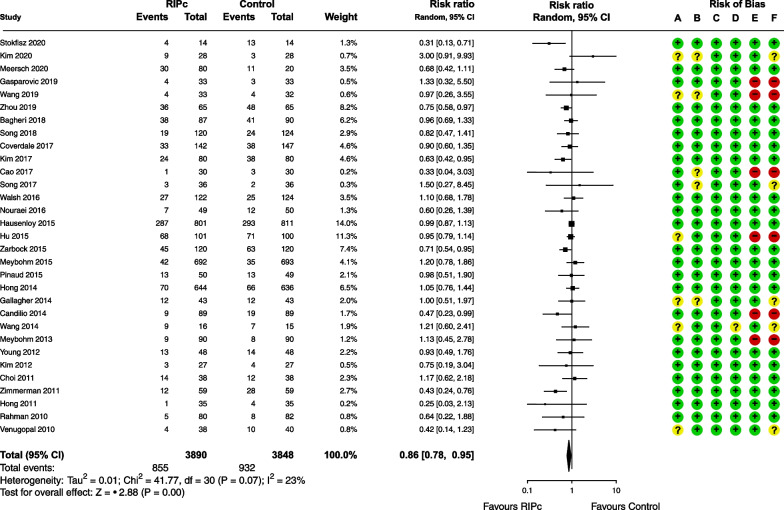
Meta-analysis of the effect of remote ischemic preconditioning (RIPc) on cardiac surgery-associated acute kidney injury. Risk of bias: **A** random sequence generation, **B** allocation concealment, **C** blinding of participants and personnel, **D** incomplete outcome data, **E** selective reporting, **F** overall bias

RIPc was associated with a significant reduction in CSA-AKI incidence as compared with sham preconditioning (RR, 0.86 [95% CI 0.78; 0.95]), with slight heterogeneity (*I*^2^ = 23%; *P*_het_ = 0.07) (Figs. 2 and 3).

Subgroup analyses by risk of bias, definition of CSA-AKI and type of surgery did not significantly change the intervention effect (Additional file 1: Figures S4-6).

The funnel plot showed a clear asymmetry in the distribution of trials, with a significant Egger’s test (*p* = 0.03) (Additional file 1: Figure S7).

##### Pulsatile flow during CPB

Among the 10 included RCTs, 5 were at low risk of bias, 4 high risk of bias and one some concerns. AKI was defined with a consensus classification (AKIN) for only one trial; 6 trials used elevated creatinine level; and 3 did not report the definition of AKI. Pulsatile flow during CPB was associated with a significant reduction in incidence of CSA-AKI (RR, 0.69 [95% CI 0.48–0.99]), with heterogeneity (*I*^2^ = 53%; *P*_het_ < 0.01) (Fig. 2; Additional file 1: Figure S8).

We performed a post-hoc subgroup analysis regarding how the pulsatile flow was performed. Among all trials, 8 used IABP and 2 pulsatile CPB. All trials using IABP involved patients with high surgical risk defined by high pre-operative score or poor left ventricular ejection fraction. The RR for IABP and pulsatile CPB were 0.66 (0.46–0.96) and 1.51 (0.16–14.27), respectively. However, the interaction test was not statistically significant as in the subgroup analyses by risk of bias and definition of CSA-AKI (Additional file 1: Figures S9-11).

We did not observe asymmetry in the funnel plot (Egger’s regression test: *p* = 0.72). (Additional file 1: Figure S12).

#### Interventions with no significant reduction in CSA-AKI incidence

##### MECC

Fourteen RCTs (1617 patients) were included, most at some concerns regarding risk of bias. AKI was defined with the AKIN classification in 5 trials and RIFLE in 1 trial. MECC was not associated with a significant reduction in CSA-AKI incidence as compared with standard CPB (RR, 0.78 [95% CI 0.54–1.13]), with low heterogeneity (*I*^2^: 0%; *P*_het_ = 0.33) (Fig. 2; Additional file 1: Figure S13). We found no change in intervention effect on subgroup analyses (Additional file 1: Figures S14-16) and no asymmetry in the funnel plot (Additional file 1: Figure S17).

##### Red blood cell (RBC) transfusion strategies

Six RCTs evaluated a restrictive versus liberal transfusion strategy (8289 patients). Three trials were at low risk of bias and 2 high risk of bias. The definition used for AKI was AKIN, KDIGO or RIFLE for 4 trials. A restrictive transfusion strategy was not associated with a significant reduction in CSA-AKI incidence as compared with a liberal transfusion strategy (RR, 1.02 [95% CI 0.92; 1.12), and no heterogeneity was detected (*I*^2^ = 3%; *P*_het_ = 0.67) (Fig. 2; Additional file 1: Figures S18-20).

##### Tight glycemic control

Ten RCTs compared tight versus standard glycemic control (2753 patients). Five trials were at low risk of bias. Only one trial used a consensus definition of AKI. Tight glycemic control was not associated with a significant reduction in CSA-AKI incidence as compared with standard glycemic control (RR, 0.86 [95% CI 0.55; 1.35]), with slight heterogeneity (*I*^2^ = 26%; *P*_het_ = 0.25) (Fig. 2; Additional file 1: Figures S21-24).

##### Other interventions

Forest plots of other interventions are in Additional file 1: Figures S25-33.

### Secondary outcomes

No intervention was associated with a significant reduction in mortality (Additional file 1: Figure S34). The RR was 1.02 (95% CI 0.76–1.38) for RIPc and 0.79 (0.51–1.23) for pulsatile flow during CPB. Nor was any intervention associated with a significant reduction in RRT use, particularly RIPc (RR, 1.00 [95% CI 0.60; 1.67]) and pulsatile flow during CPB (RR, 0.67 [95% CI 0.39–1.15]) (Additional file 1: Figure S35).

The only intervention with a potential effect on reducing the hospital stay was the use of pulsatile blood flow during CPB (MD, − 0.74 [95% CI − 0.85 to − 0.63]), with moderate heterogeneity (*I*^2^ = 52%; *P*_het_ = 0.04) (Additional file 1: Figure S36). Pulsatile blood flow was associated with reduced ICU stay (MD, − 0.39 [− 0.50 to − 0.27]; *I*^2^ = 90%, *P*_het_ < 0.01), as was MECC (MD, − 0.49 [− 0.60 to − 0.24]; *I*^2^ = 85%, *P*_het_ < 0.01) (Additional file 1: Figure S37). The use of MECC was also associated with a significantly reduced stroke incidence (RR, 0.36 [95% CI 0.15; 0.86]), with no heterogeneity detected (*I*^2^ = 0%; *P*_het_ = 0.89). No other intervention was associated with a reduction in stroke incidence (Additional file 1: Figure S38)*.* No intervention had a significant effect on myocardial infarction or atrial fibrillation (Additional file 1: Figures S39-40)*.*

### Quality of evidence

**Fig. 4:**
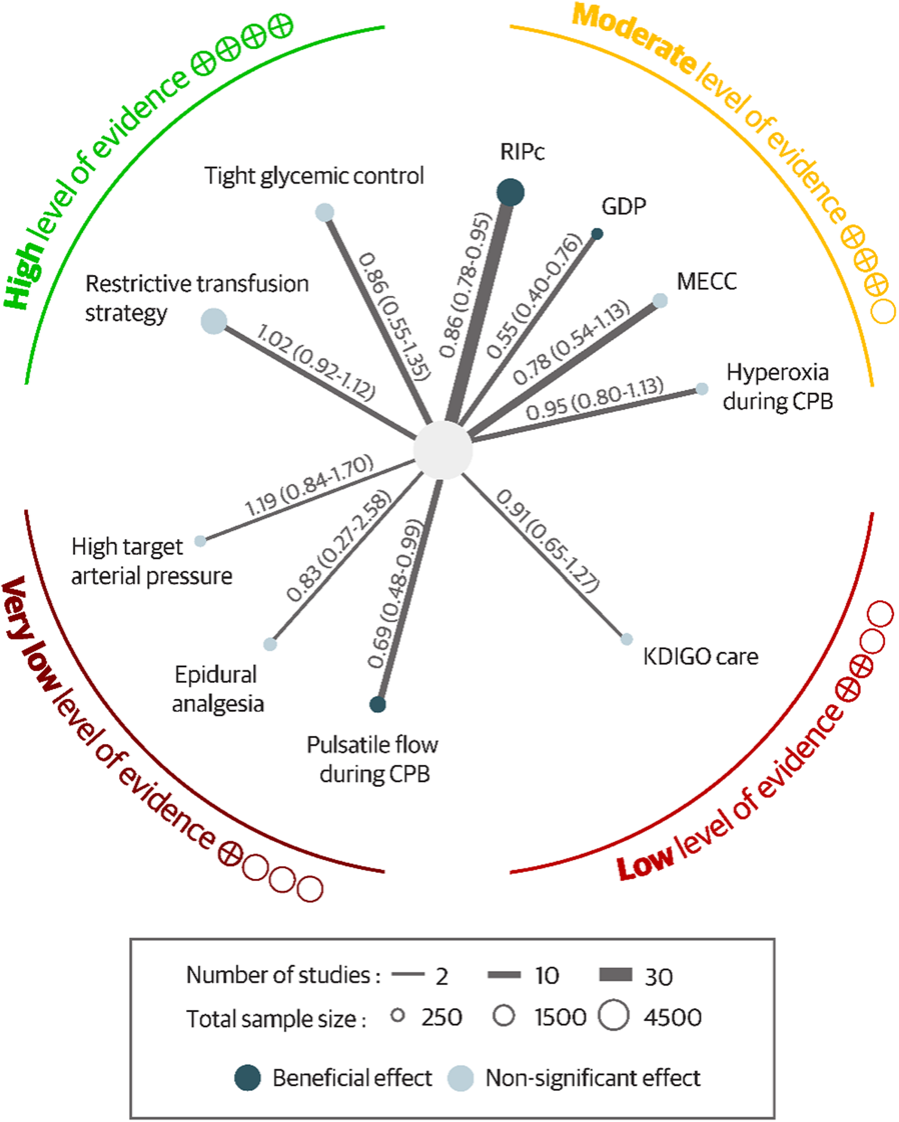
Quality of evidence of each non-pharmacological intervention for preventing cardiac surgery-associated acute kidney injury. CPB, cardiopulmonary bypass; KDIGO, Kidney Disease Improving Global Outcomes; RIPc, Remote ischemic preconditioning; GDP, Goal Directed Perfusion; MECC, Minimally invasive extracorporeal circulation

Among the 3 interventions showing a significantly reduced CSA-AKI incidence, none exhibited a high quality of evidence. GDP and RIPc had moderate quality of evidence related to the low number of trials and a small study effect, respectively, whereas pulsatile flow during CPB had very low quality of evidence due to heterogeneity and high risk of bias in included trials. Two interventions (RBC transfusion strategy and tight glycemic control) exhibited high quality of evidence for the lack of benefit on reducing the incidence of CSA-AKI (Fig. 4, Additional file 1: Table S4).

## Discussion

Our meta-analysis identified 10 non-pharmacological interventions to reduce CSA-AKI. We found the use of GDP, RIPc and pulsatile blood flow during CPB associated with significantly reduced incidence of CSA-AKI. However, no intervention had high quality of evidence. Conversely, 2 interventions (restrictive transfusion strategy and tight glycemic control) had high quality of evidence for a lack of effect on reducing CSA-AKI incidence. Although we restricted our search to recent trials, the definition of CSA-AKI was heterogeneous across trials with nearly half of the trials using a non-consensus definition of AKI, despite a trend to increased use of a consensus definition of AKI over the years.

This is the first meta-analysis synthesizing all non-pharmacological interventions during the peri-operative period to reduce the risk of CSA-AKI. Our search strategy was extensive, without language restriction, thus limiting the risk of missing important trials, with a robust and standardized methodology based on the Cochrane Handbook including the GRADE approach for evaluating the certainty of evidence. The protocol was prospectively registered in PROSPERO. Our meta-analysis brings new insights into the prevention of CSA-AKI, with important implications for clinical practice and future research. We provide a comprehensive summary of non-pharmacological interventions for prevention of CSA-AKI including an evaluation of their level of evidence. While recent meta-analyses focused on specific interventions such as RIPc, pulsatile flow during CPB or GDP, no previous meta-analysis has concentrated on CSA-AKI prevention for the following interventions: red blood cell transfusion strategies, tight glycemic control, minimally invasive extracorporeal circulation, epidural analgesia, KDIGO care, targeting high-arterial pressure or hyperoxia during CPB.

In our meta-analysis, GDP was significantly associated with reduced incidence of CSA-AKI. GDP is a recent intervention [27], derived from the goal-directed therapy used in the care of critically ill patients [28]. It is based on directly monitoring the oxygen delivery (DO2) during CPB, thus allowing the perfusionist to target above a critical threshold of DO2, usually 280 ml/min/m2. In practice, the DO2 target can be achieved by an increase in pump flow or RBC transfusion. In one of the trials included in our meta-analysis [29], DO2 was achieved only by pump-flow adjustment. Moreover, in the other trial [30], RBC transfusion rate did not differ between the 2 groups, which suggests that optimization of DO2 is mainly achieved by adjusting pump flow. A recent meta-analysis on GDP including both RCTs and an observational study, also found a beneficial effect on prevention of CSA-AKI, especially for AKI stage 1 but not for stages 2 or 3 [31]. While focusing on RCTs, our results support this with a moderate quality of evidence because only two RCTs have been performed.

RIPc was the most evaluated intervention in our meta-analysis, with more than 30 trials, and was associated with decreased incidence in CSA-AKI. RIPc is a simple, non-invasive and inexpensive strategy. During the last 2 decades, several trials and meta-analyses assessing RIPc in cardiac surgery were published, with discordant results. In 2015, 2 large multicenter RCTs (ERRICCA [32] and RIPHeart [33]) failed to show a clinical benefit of RIPc in reducing the incidence of CSA-AKI. Several hypotheses could explain the discrepancies. First, they could be related to a small study effect, the tendency for small trials to show larger treatment effects than larger trials within a meta-analysis [34] and reflecting dissemination bias. In our meta-analysis, we found a clear asymmetry in the funnel plot, thus suggesting a small study effect. We used random-effects models, which give more weight to small studies and may overestimate the treatment effect. In a sensitivity post-hoc analysis using a fixed-effects model, we found consistent results, with significant results for RIPc, but we still cannot exclude a possible impact of the small study effect on our result. Another possible cause of discrepancies may be the population included in the 2 large RCTs, with high-risk patients in the ERICCA trial or conversely low-risk patients in the RIPHeart trial. In our meta-analysis, we included all patients undergoing cardiac surgery, with no restriction on risk. Third, general anesthesia mainly involved propofol in both trials; however, some data suggest that propofol may inhibit the cardioprotective effect of RIPc [35] and reduce the impact of RIPc on CSA-AKI. Finally, the different modalities of RIPc could explain the discrepancies. In both ERICCA and RIPHeart trials, RIPc was applied to the arm, whereas a clinical study showed a larger benefit on endothelial ischemia–reperfusion injury when RIPc was applied to the leg than the arm [36].Two previous meta-analyses assessing RIPc [37, 38] showed results consistent with our finding. However, neither of these studies evaluated the quality of evidence. RIPc is not recommended in current practice guideline due to insufficient strength of evidence [39]. In our meta-analysis we found a benefice of RIPc on reduction of CSA-AKI with a moderate level of evidence related to the small study effect, highlighting the need of further RCTs to confirm this benefit.

Laminar flow during CPB affects microcirculatory perfusion via endothelial damage [40, 41], which could alter renal perfusion. In this context, pulsatile flow seems more natural than laminar flow in CPB, and physiological studies found that it can improve microcirculatory parameters [42] and oxygen extraction and decrease systemic vascular resistance [43]. Pulsatile flow during CPB can be performed by two 2 means: directly by the CPB machine via the blood pump or with an IABP. Pulsatile blood flow delivered by the CPB, essentially used for low-risk patients, had no significant benefit for CSA-AKI, but the use of IABP in high-risk patients significantly reduced the incidence of CSA-AKI. IABP is a cardiac assistance with potential harmful effects [44] and therefore cannot be proposed for all patients. During the last decade, two meta-analyses assessing pulsatile flow during CPB [45, 46], showed a potential beneficial effect for CSA-AKI prevention. However, they did not focus on RCTs only, were not registered on PROSPERO and did not assess risk of bias. In our meta-analysis, we found that pulsatile blood flow could reduce CSA-AKI but with a very low quality of evidence related to high heterogeneity and risk of bias in trials. This very low quality of evidence highlights the need of conducting further RCTs for this intervention.

The other interventions did not have a significant effect on reducing the incidence of CSA-AKI. Some interventions exhibited very low or low level of evidence, such as the use of epidural analgesia or KDIGO care bundle; therefore, more trials are needed to assess their usefulness to reduce the incidence of CSA-AKI. In contrast, our meta-analysis is the first to show a high quality of evidence for the lack of effect of restrictive transfusion strategy and tight glycemic control for preventing CSA-AKI. Consistent with our results, 2 recent meta-analyses showed a lack of benefit of a restrictive transfusion strategy in cardiac surgery [47, 48]. Given these findings, further trials assessing these interventions are unnecessary.

No intervention was associated with a reduction in need for RRT after cardiac surgery, which may be due to the low proportion of patients requiring RRT and the lack of reporting of this outcome in some trials.

Our results highlight several issues with important implications regarding studies of CSA-AKI. First, a large number of trials did not report CSA-AKI in their outcomes and were therefore excluded. Hence, our results, based on secondary outcomes, may not be exhaustive because some trials may have assessed some of our secondary outcomes without including CSA-AKI among their outcomes. The number of RCTs of cardiac surgery that did not consider AKI in their outcomes was still high, with 40% of trials excluded for this reason. Although AKI remains one of the most common complications [49] with a high impact on short- and long-term survival after cardiac surgery [50], it is not part of the core outcome set for adult cardiac surgery trials [51]. Because AKI is systematically assessed after cardiac surgery, it seems important and simple to consider that outcome for future trials. Second, despite having restricted our selection to a recent period with a consensus, definition of AKI remained heterogeneous across trials including the most recent ones. The first consensus definition of AKI was proposed in 2004 (RIFLE), with an update in 2007 (AKIN) and 2012 (KDIGO). However, we included trials published from 2004 that were planned before this date. The proportion of consensus definitions of AKI increased in recent trials, which is encouraging but remains insufficient. Only 65% of the trials published in the last 5 years reported a consensus definition for AKI. Therefore, we highlight the importance of using a consensus definition of AKI in trials [52].

From a comprehensive perspective, considering the growing utilization of multimodal.

protective strategies in cardiac surgery [53, 54], our meta-analysis may help implementing an evidence-based care bundle that includes interventions showing a reduction in the risk of AKI, supported by at least a moderate level of evidence, such as GDP and RIPc.

In contrast, interventions with a high quality of evidence for the lack of benefit on reducing the incidence of CSA-AKI such as tight glycemic control and transfusion strategy should not be incorporated into a future bundle of care for preventing CSA-AKI.

## Conclusions

In our meta-analysis, we identified 3 non-pharmacological interventions that could reduce CSA-AKI incidence: GDP and RIPc with moderate quality of evidence and pulsatile flow during CPB with very low quality of evidence. We also identified 2 interventions (restrictive transfusion strategy and tight glycemic control) with no benefit in reducing CSA-AKI incidence and with high quality of evidence. However, many trials of cardiac surgery did not consider CSA-AKI among outcomes even though it is one of the most frequent complications after cardiac surgery. Also, when trials did consider the CSA-AKI outcome, the definition of AKI was heterogeneous across trials despite the development and validation of consensus definitions.

### Supplementary Information

**Additional file 1: eTable 1.** Changes between the protocol and the article in the Outcome and the Data Analysis. **Supplemental eMaterial 1.** Search algorithm in PubMed. **eTable2.** Detailed characteristics of each included trial by intervention. **eTable3.** Characteristics of chronic kidney disease population for each intervention. **eFigure 1.** Risk of bias of randomized controlled trials assessing a non-pharmacological intervention to prevent cardiac surgery associated - acute kidney injury. **eFigure 2a.** Definition of cardiac surgery associated acute kidney injury in all included RCTs according to each intervention. **eFigure 2b.** Acute kidney injury definition according to the year of publication of the trials. **eFigure 3.** Meta-analysis of the effect of goal directed perfusion (GDP) on cardiac surgery associated acute kidney injury. **eFigure 4.** Subgroup analysis of the effect of Remote ischemic preconditioning (RIPc) on cardiac surgery associated acute kidney injury according the Risk of bias. **eFigure 5.** Subgroup analysis of the effect of Remote ischemic preconditioning (RIPc) on cardiac surgery associated acute kidney injury according the definition of acute kidney injury (AKI). **eFigure 6.** Subgroup analysis of the effect of Remote ischemic preconditioning (RIPc) on cardiac surgery associated acute kidney injury according the type of surgery. **eFigure 7.** Funnel plot for random effects meta-analysis of cardiac surgery associated acute kidney injury outcomes in trials of Remote ischemic preconditioning. **eFigure 8.** Meta-analysis of the effect of pulsatile flow on cardiac surgery associated acute kidney injury. **eFigure 9.** Subgroup analysis of the effect of pulsatile flow on cardiac surgery associated acute kidney injury according the modality of pulsatility. **eFigure 10.** Subgroup analysis of the effect of pulsatile flow on cardiac surgery associated acute kidney injury according the definition of acute kidney injury (AKI). **eFigure 11.** Subgroup analysis of the effect of pulsatile flow on cardiac surgery associated acute kidney injury according the risk of bias. **eFigure 12.** Funnel plot for random effects meta-analysis of CSA-AKI outcomes in RCTs of Pulsatile flow during CPB. **eFigure 13.** Meta-analysis of the effect of Minimally invasive extracorporeal circulation (MECC) on cardiac surgery associated acute kidney injury. **eFigure 14.** Subgroup analysis of the effect of Minimally invasive extracorporeal circulation (MECC) on cardiac surgery associated acute kidney injury according the risk of bias. **eFigure 15.** Subgroup analysis of the effect of Minimally invasive extracorporeal circulation (MECC) on cardiac surgery associated acute kidney injury according the definition of acute kidney injury (AKI). **eFigure 16.** Subgroup analysis of the effect of Minimally invasive extracorporeal circulation (MECC) on cardiac surgery associated acute kidney injury according the type of surgery. **eFigure 17.** Funnel plot for random effects meta-analysis of cardiac surgery associated acute kidney injury outcomes in trials of Minimally invasive extracorporeal circulation (MECC). **eFigure 18.** Meta-analysis of the effect of restrictive transfusion strategy on cardiac surgery associated acute kidney injury. **eFigure 19.** Subgroup analysis of the effect of restrictive transfusion strategy on cardiac surgery associated acute kidney injury according the risk of bias. **eFigure 20.** Subgroup analysis of the effect of restrictive transfusion strategy on cardiac surgery associated acute kidney injury according the definition of acute kidney injury (AKI). **eFigure 21.** Meta-analysis of the effect of tight glycemic (BG) control on cardiac surgery associated acute kidney injury. **eFigure 22.** Subgroup analysis of the effect of tight glycemic (BG) control on cardiac surgery associated acute kidney injury according the risk of bias. **eFigure 23.** Subgroup analysis of the effect of tight glycemic (BG) control on cardiac surgery associated acute kidney injury according the definition of acute kidney injury (AKI). eFigure 24. Funnel plot of tight glycemic control on cardiac surgery associated acute kidney injury. **eFigure 25.** Meta-analysis of the effect of Epidural analgesia on cardiac surgery associated acute kidney injury. **eFigure 26.** Subgroup analysis of the effect of Epidural analgesia on cardiac surgery associated acute kidney injury according the risk of bias. **eFigure 27.** Subgroup analysis of the effect of Epidural analgesia on cardiac surgery associated acute kidney injury according the definition of acute kidney injury (AKI). **eFigure 28.** Meta-analysis of the effect of KDIGO bundle of care on cardiac surgery associated acute kidney injury. **eFigure 29.** Subgroup analysis of the effect of KDIGO care on cardiac surgery associated acute kidney injury according the risk of bias. **eFigure 30.** Meta-analysis of the effect of high-target arterial pressure (MAP) target on cardiac surgery associated acute kidney injury. **eFigure 31.** Subgroup analysis of the effect of high-target arterial pressure (MAP) target on cardiac surgery associated acute kidney injury according the definition of acute kidney injury (AKI). **eFigure 32.** Meta-analysis of the effect of hyperoxia on cardiac surgery associated acute kidney injury. **eFigure 33.** Subgroup analysis of the effect of hyperoxia on cardiac surgery associated acute kidney injury according the risk of bias. **eFigure 34.** Forest plot of non-pharmacological interventions for efficacy in reducing risk of mortality. Interventions were tested with standard medical care as control. **eFigure 35.** Forest plot of non-pharmacological interventions for efficacy in reducing risk of RRT. Interventions were tested with standard medical care as control. **eFigure 36.** Forest plot of non-pharmacological interventions for efficacy in reducing lenght of hospital stay. Interventions were tested with standard medical care as control. **eFigure 37.** Forest plot of non-pharmacological interventions for efficacy in reducing length of Intensive care unit stay. Interventions were tested with standard medical care as control. **eFigure 38.** Forest plot of non-pharmacological interventions for efficacy in reducing risk of post-operative stroke. Interventions were tested with standard medical care as control. **eFigure 39.** Forest plot of non-pharmacological interventions for efficacy in reducing risk of post-operative myocardial infarction. Interventions were tested with standard medical care as control. **eFigure 40.** Forest plot of non-pharmacological interventions for efficacy in reducing risk of post-operative atrial fibrillation. Interventions were tested with standard medical care as control. **eTable 4.** Summary of quality of evidence for the effect of each intervention on CSA-AKI according to the Grading of Recommendations Assessment, Development, and Evaluation (GRADE).

## Data Availability

Data available: Yes. Data types: Data (not involving human participants). How to access data: geoffroy.hariri@aphp.fr. When available: With publication. *Supporting Documents* Document types: Statistical/analytic code. How to access documents: geoffroy.hariri@aphp.fr. When available: With publication. *Additional Information* Who can access the data: anyone requesting the data. Types of analyses: for a specified purpose. Mechanisms of data availability: with a signed data access agreement.

## Abbreviations

AKI: Acute kidney injury
AKIN: Acute Kidney Injury Network
CABG: Coronary artery bypass grafting
CIs: Confidence intervals
CPB: Cardiopulmonary bypass
CKD: Chronic kidney disease
CSA-AKI: Cardiac surgery-associated acute kidney injury
DO2: Oxygen delivery
GDP: Goal-directed perfusion
GRADE: Grading of Recommendation, Assessment, Development, and Evaluation
IABP: Intra-aortic balloon pump
ICU: Intensive care unit
IQR: Interquartile range
KDIGO: Kidney Disease Improving Global Outcomes
MD: Mean differences
MECC: Minimally invasive extracorporeal circuit
RBC: Red blood cell
RCTs: Randomized controlled trials
RIPc: Remote ischemic preconditioning
RRs: Risk ratios
RRT: Renal replacement therapy
SD: Standard deviation
