# Supplementary material for: Prevention of cardiac surgery-associated acute kidney injury: a systematic review and meta-analysis of non-pharmacological interventions
Source: Crit Care. 2023 Sep 12;27:354. doi: 10.1186/s13054-023-04640-1 (PMC10498585; doi:10.1186/s13054-023-04640-1)
Supplement: Supplementary file 1 — Additional file 1: eTable 1. Changes between the protocol and the article in the Outcome and the Data Analysis. Supplemental eMaterial 1. Search algorithm in PubMed. eTable2. Detailed characteristics of each included trial by intervention. eTable3. Characteristics of chronic kidney disease population for each intervention. eFigure 1. Risk of bias of randomized controlled trials assessing a non-pharmacological intervention to prevent cardiac surgery associated - acute kidney injury. eFigure 2a. Definition of cardiac surgery associated acute kidney injury in all included RCTs according to each intervention. eFigure 2b. Acute kidney injury definition according to the year of publication of the trials. eFigure 3. Meta-analysis of the effect of goal directed perfusion (GDP) on cardiac surgery associated acute kidney injury. eFigure 4. Subgroup analysis of the effect of Remote ischemic preconditioning (RIPc) on cardiac surgery associated acute kidney injury according the Risk of bias. eFigure 5. Subgroup analysis of the effect of Remote ischemic preconditioning (RIPc) on cardiac surgery associated acute kidney injury according the definition of acute kidney injury (AKI). eFigure 6. Subgroup analysis of the effect of Remote ischemic preconditioning (RIPc) on cardiac surgery associated acute kidney injury according the type of surgery. eFigure 7. Funnel plot for random effects meta-analysis of cardiac surgery associated acute kidney injury outcomes in trials of Remote ischemic preconditioning. eFigure 8. Meta-analysis of the effect of pulsatile flow on cardiac surgery associated acute kidney injury. eFigure 9. Subgroup analysis of the effect of pulsatile flow on cardiac surgery associated acute kidney injury according the modality of pulsatility. eFigure 10. Subgroup analysis of the effect of pulsatile flow on cardiac surgery associated acute kidney injury according the definition of acute kidney injury (AKI). eFigure 11. Subgroup analysis of the effect of pulsatile flow on [file 13054_2023_4640_MOESM1_ESM.docx]

**Supplemental Material**

**eTable 1. Changes between the protocol and the article in the Outcome and the Data Analysis**

| **Protocol** | **Article** | **Justification** |
| --- | --- | --- |
| **Outcome** |  |  |
| -Acute kidney injury (AKI) within 7 days after cardiac surgery defined by at least one of the following diagnostic criteria: risk, injury, failure, loss of function and end- stage renal disease (RIFLE); Acute Kidney Injury Network (AKIN); and kidney disease improving global outcomes (KDIGO) criteria | Post-operative AKI according to all definitions | Many trials assessing AKI without a consensual classification |
| -Serum creatinine at H24 | Not reported | No data |
| -Length of mechanical ventilation and catecholamines administration | Not reported | No data |
| -ICU mortality/ all-cause hospital mortality | Combined in “hospital mortality” | Mainly reported as ‘in hospital mortality” in the included trials |
| -All adverse event and serious adverse event | Not reported | No data |
| **Data Analysis** |  |  |
| -Comparisons via a network meta-analysis (NMA) | Conventional meta-analyses for direct comparisons for each intervention | All RCTs compared the experimental intervention versus usual care and there was no comparison between different interventions |
| -Subgroup analysis according AKIN, KDIGO, RIFLE | Subgroup analysis according to all definitions of AKI | Many trials assessing AKI without a consensual classification |

AKI: Acute kidney injury. RIFLE: risk, injury, failure, loss of function and end- stage renal disease. AKIN: Acute Kidney Injury Network. KDIGO: kidney disease improving global outcomes. RCT: Randomized controlled trial. ICU: Intensive care unit.

**Supplemental eMaterial 1. Search algorithm in PubMed**

#1 "cardiac surgical procedures"[MeSH Terms]

#2 "cardiopulmonary bypass"[MeSH Terms]

#3 "cardiothoracic surgery" [Title]

#4 "cardiac surgeries" [Title]

#5 "cardiac surgery"[Title]

#6 "cardiovascular surgery"[Title]

#7 "coronary artery bypass graft"[Title]

#8 "valve replacement"[Title]

#9 "cardiopulmonary bypass"[Title]

#10 "mitral valve"[Title]

#11 "aortic valve"[Title]

#12 "heart valve"[Title]

#13 "heart surgery"[Title]

#14 "valve repair"[Title]

#15 #1 OR #2 OR #3 OR #4 OR #5 OR #6 OR #7 OR #8 OR #9 OR #10 OR #11 OR #12 OR #13 OR #14

#16 "transcatheter"[Title/Abstract]

#17 "mitraclip"[Title]

#18"percutaneous "[Title]

#19 “heart transplantation” [Title]

#20 "cardiac transplantation"[Title]

#21 #16 OR #17 OR #18 OR #19 OR 20

#22 #15 NOT #21

#23 "acute kidney injury"[MeSH Terms]

#24 "acute kidney injury"[Title/Abstract]

#25 "creatinine"[Title/Abstract]

#26 "acute kidney failure"[Title/Abstract]

#27 "acute renal dysfunction" [Title/Abstract]

#28 "acute renal insufficiency"[Title/Abstract]

#29 "acute renal injury" [Title/Abstract]

#30 "AKI" [Title/Abstract]

#31 "kidney" [Title/Abstract]

#32 "renal" [Title/Abstract]

#33 "renal failure" [Title/Abstract]

#34 "renal replacement therapy" [Title/Abstract]

#35 #23 OR #24 OR #25 OR #26 OR #27 OR #28 OR #29 OR #30 OR #31 OR #32 OR #33 OR #34

#36 "randomized controlled trial"[Publication Type]

#37 "controlled clinical trial"[Publication Type]

#38 "randomized"[Title/Abstract]

#39 "placebo"[Title/Abstract]

#40 "randomly"[Title/Abstract]

#41 "trial"[Title/Abstract]

#42 "groups"[Title/Abstract]

#43 "drug therapy"[MeSH Subheading]

#44 #36 OR #37 OR #38 OR #39 OR #40 OR #41 OR #42 OR #43

#45 "animals"[MeSH Terms] NOT "human"[MeSH Terms]

#46 "pediatric"[Title]

#47 "child*"[Title]

#48 "paediatric"[Title]

#49 "infant*"[Title]

#50 "neonate*"[Title]

#51 #45 OR #46 OR #47 OR #48 OR #49 OR #50

#52 #44 NOT #51

#53 #22 AND #35 AND #52

**eTable2.** **Detailed characteristics of each included trial by intervention**

| Trial | Design | Nb of centers | Countries | Funding | Recruitment period | Population | Intervention | Control | Primary outcome | AKI definition |
| --- | --- | --- | --- | --- | --- | --- | --- | --- | --- | --- |
| Goal directed perfusion | | | | | | | | | | |
| Mukaidai 2021 | Superiority | 1 | Japan | No | 2018-2020 |  | DO2>300mL/min | Usual care | AKI | KDIGO |
| Ranucci 2019 | Superiority | 9 | Europe, Australia, New Zealand, U.S | Mixed | 2014-2017 | predicted CBP> 90 min | DO2>280ml/min | Usual care | AKI | AKIN |
| Remote Ischemic Preconditioning | | | | | | | | | | |
| Bagheri 2018 | Superiority | 1 | Iran | Public | 2013-2017 | On-pump CABG | After induction 3x5min 200mmHg. Arm | Usual care | AKI | AKIN |
| Candilio 2014 | Superiority | 1 | UK | Mixed | 2010-2012 | On-pump CABG | After induction 2x 5min. 200mmHg or >15mmHg. 2 Arms | Usual care | HsTnT | AKIN |
| Cao 2017 | Parallel | 1 | China | NR | 2016-2016 | Valve surgery | After induction 3x5min 200mmHg. Leg | Usual care | CK-MB | NR |
| Choi 2011 | Superiority | 1 | Korea | NR | 2008-2009 | Valve or combined surgery | After induction 3x5min 250mmHg. Leg | Usual care | AKI | AKIN |
| Coverdale 2017 | Superiority | 1 | Canada | NR | 2012-2015 | On-pump  CKD LVEF<40% | Pre-operative 3x5min 200mmHg. Arm | Usual care | Composite: all-cause mortality, MI, stroke, respiratory failure, acute renal failure, and LCOS | NR |
| Gallagher 2014 | Superiority | 1 | UK | Mixed | 2011-2012 | On-pump CABG or combined surgery  CKD | After induction 3x5min >50mmHg SAP Arm | Usual care | AKI | AKIN |
| Gasparovic 2019 | Superiority | 1 | Croatia | Public | 2014-2015 | On-pump CABG | After induction 3x5min 200mmHg. Arm | Usual care | New brain ischemia on structural MRI and new impairment in neurocognition | RIFLE |
| Hausenloy 2015 | Superiority | 30 | UK | Mixed | 2011-2014 | On-pump. CABG or combined. Euroscore >5 | After induction. 4x5min 200mmHg (or>15mmHg). Arm | Usual care | Death from cardiovascular causes, nonfatal MI, coronary revascularization, or stroke | KDIGO |
| Hong 2011 | Superiority | 1 | Korea | Mixed | NR | On-pump. CABG | 2 times: After induction and after last bypass 4x5min 200mmHg.  Leg | Usual care | Myocardial injury reduction | creatinine>2mg/dl with increase of >0,7 from baseline |
| Hong 2014 | Superiority | 2 | Korea | Public | 2009-2010 | On-pump | 2 times: After induction and after CPB 4x5min 200mmHg. Arm 4x5min  200mmHg Arm | Usual care | Composite: death, MI, arrhythmia requiring treatment, stroke, coma, renal failure, respiratory failure, cardiogenic shock, GI complication, and MOF | creatinine>2mg/dl with increase of >0,7 from baseline |
| Hu 2015 | Superiority | 2 | China | Mixed | 2004-2005 | Valve surgery | After aortic cross clamp 3x5min 600mmHg. Leg | Usual care | Cardiac troponin | AKIN |
| Kim 2012 | Superiority | 1 | Korea | NR | 2010-2011 | Combined surgery | After induction and after CPB weaning 3x10min 250mmHg. Leg | Usual care | PaO2/FiO2 | Increased creatinine >50% or 0,3mg/dL |
| Kim 2017 | Superiority | 1 | Korea | NR | 2013-2015 | On-pump | Pre-operative 4x5min 200mmHg. Arm | Usual care | Troponin I | AKIN |
| Kim 2020 | Superiority | 1 | Korea | Public | 2017-2019 | Off-pump CABG | After induction 4x5min 200mmHg. Arm | Usual care | ADP test | KDIGO |
| Meersch 2020 | NR | 1 | Germany | Mixed | 2016-2018 | On-pump Cleveland score>6 | Multiarm | Usual care | urinary [TIMP2]*[IGFBP7] | KDIGO |
| Meybohm 2013 | Superiority | 1 | Germany | Public | 2009-2010 | On-pump CABG | After induction 4x5min 200mmHg. Leg | Usual care | Postoperative neurocognitive dysfunction | AKIN |
| Meybohm 2015 | Both | 14 | Germany | Public | 2007-2009 | CABG Diabetic or BG>150 | After induction 4x5min 200mmHg (or>15mmHg). Arm | Usual care | Death from any cause, nonfatal MI, new stroke, or acute renal failure up to the time of hospital discharge | RIFLE |
| Nouraei 2016 | Superiority | 1 | Iran | NR | 2013-2015 | On-pump CABG | After induction 3x5min >20mmHg SAP. Leg | Usual care | AKI | AKIN |
| Pinaud 2015 | Superiority | 1 | France | Public | 2011-2012 | Valve surgery | After induction 3x5min 200mmHg. Arm | Usual care | Troponin I | AKIN |
| Rahman 2010 | Superiority | 1 | UK | Public | 2007-2009 | On-pump CABG | After induction 3x5min 200mmHg. Arm | Usual care | Troponin T | increased creatinine >0,5mg/dl |
| Song 2017 | Superiority | 1 | Korea | Public | NR | Valve surgery Patients with myocardial hypertrophy | After induction 3x5min 200mmHg. Arm | Usual care | CK-MB and troponin I | AKIN |
| Song 2018 | Superiority | 1 | Korea | NR | 2016-2017 | Valve surgery | Aortic declamping /H12/H24. 3x5min on the thigh | Usual care | AKI | KDIGO |
| Stokfisz 2020 | Superiority | 1 | Poland | NA | 2014-2014 | Off-pump CABG | After induction 3x5min 200mmHg | Usual care | AKI | KDIGO |
| Venugopal 2010 | Parallel | 1 | UK | Public | NR | On-pump CABG Non-diabetic | 3x5min 200mmHg. Arm | Usual care | AKI | AKIN |
| Walsh 2016 | NR | 4 | USA, Canada | Mixed | 2011-2012 | On-pump CABG Euroscore >6 | Before CPB 2x5min 300mmHg. Leg | Usual care | CK-MB | AKIN |
| Wang 2014 | NR | 1 | China | NR | 2011-2011 | Valve replacement | After induction 4x5min 600mmHg. Leg | Usual care | NGAL | AKIN |
| Wang 2019 | Superiority | 1 | China | Private | NR | Off-pump CABG | After induction 4x5min >40mmHg SAP. Leg | Usual care | Myocardial injury | NR |
| Young 2012 | Superiority | 1 | New-Zealand | Public | 2010-2011 | On-pump CABG | After incision 3x5min 200mmHg. Leg | Usual care | Troponin T | RIFLE |
| Zarbock 2015 | Superiority | 4 | Germany | Public | 2013-2014 | On-pump | After induction 3x5min 200mmHg Arm | Usual care | AKI | KDIGO |
| Zhou 2019 | Superiority | 1 | China | No | 2017-2018 | Total arch replacement | After induction 3x5min 200mmHg (or>50mmHg). Arm | Usual care | AKI | KDIGO |
| Zimmerman 2011 | Superiority | 1 | USA | NR | 2008-2009 | On-pump CABG | After induction 3x5min 200mmHg. Leg | Usual care | AKI | AKIN |
| Pulsatile flow during CPB | | | | | | | | | | |
| Adademir 2012 | NR | 1 | Turkey | NR | 2009-2009 | On-pump CABG | Pulsatile CPB | Usual care | Kidney function tests | Increased creatinine>50% |
| Ferreira 2018 | Superiority | 1 | Brazil | NR | 2014-2016 | On-pump CABG or combined surgery LVEF<40% | IABP | Usual care | Mortality and major postoperative complications | AKIN |
| Graßler 2019 | Superiority | 1 | Germany | NR | NA | On-pump CABG | Pulsatile CPB | Usual care | Difference in the PAI-1:tPA ratio | NR |
| Onorati 2007 | NR | 1 | Italy | NR | 2004-2006 | On-pump CABG LVEF>35% | IABP | Usual care | Renal function | Increased creatinine>50% |
| Onorati 2009 | NR | 1 | Italy | NR | 2003-2008 | On-pump CABG LVEF<40% | IABP | Usual care | Perioperative changes in creatinine clearance | KDOQI |
| Onorati 2009 | NR | 1 | Italy | NR | 2003-2007 | On-pump CABG LVEF<40% Elderly >70yo | IABP | Usual care | Perioperative changes of creatinine clearance | Increased creatinine>50% |
| Ranucci 2013 | Superiority | 1 | Italy | NR | 2009-2012 | On-pump CABG or combined surgery LVEF<35% | IABP | Usual care | Major morbidity | RRT or creatinine  increase > 2mg/dL and > 2-fold |
| Serraino 2012 | Superiority | 1 | Italy | NR | 2004-2010 | On-pump CABG LVEF<40% | IABP | Usual care | Renal function | 50% increased creatinine |
| Shi 2011 | Non-inferiority | 1 | China | NR | 1999-2010 | Off-pump CABG in acute MI with LVEF<40% | IABP | Usual care | Clinical benefit | NR |
| Wilczyński 2010 | Non-inferiority | 1 | Poland | NR | 2004-2008 | On-pump CABG LVEF<35% | IABP | Usual care | MACCE | NR |
| Minimally invasive extracorporeal circulation | | | | | | | | | | |
| Anastasiadis 2016 | NR | 1 | Greece | NR | 2007-NR | On-pump CABG | MECC | Usual care | Learning curve | AKIN |
| Asteriou 2013 | Superiority | 1 | Greece | NR | 2010-2012 | On-pump CABG | MECC | Usual care | Major adverse events | increased creatinine>2 mg/dL or twice preoperative creatinine |
| Beghi 2006 | NR | 1 | Italy | NR | 2001-2002 | On-pump CABG LVEF>40% | MECC | Usual care | Operative mortality | increased of creatinine>2mg/dl |
| Camboni 2009 | NR | 1 | Germany | NR | 2005-2006 | On-pump CABG | MECC | Usual care | Mortality | increased of creatinine>2mg/dl or RRT |
| Chew 2016 | Superiority | 1 | Singapore | Public | 2009-2012 | On-pump CABG | MECC | Usual care | AKI | AKIN |
| Deninger 2015 | Superiority | 1 | Germany | Private | 2013-2014 | On-pump CABG | MECC | Usual care | Kidney function | AKIN |
| Halfwerk 2019 | Superiority | 1 | Netherland | Public | 2012-2016 | Valve surgery | MECC | Usual care | postoperative blood loss | RIFLE |
| Kiessling 2018 | Superiority | 1 | Germany | NR | 2013-2017 | On-pump CABG | MECC | Usual care | Composite of mortality, MI, AF, renal RRT | NR |
| Media 2020 | Superiority | 1 | Denmark | Mixed | 2017-2018 | On-pump CABG | MECC | Usual care | AKI | AKIN |
| Ng 2015 | NR | 1 | Singapore | Public | 2009-2012 | On-pump CABG | MECC | Usual care | Inflammatory markers | AKIN |
| Ohata 2008 | NR | 1 | Japan | NR | 2002-2006 | On-pump CABG | MECC | Usual care | Inflammatory markers | increased of creatinine>2mg/dl |
| Remadi 2004 | Parallel | 1 | France | NR | 2001-2002 | Valve replacement | MECC | Usual care | Operative mortality | RRT |
| Remadi 2006 | Superiority | 1 | France | NR | 1998-2003 | On-pump CABG | MECC | Usual care | Operative mortality | NR |
| Rimpiläinen 2011 | Superiority | 1 | Finland | No | 2007-2009 | Valve or combined surgery | MECC | Usual care | Change in retinal micro embolic count | 1,5-fold creatinine increase |
| Epidural analgesia | | | | | | | | | | |
| Greisen 2013 | Superiority | 1 | Denmark | NR | NR |  | 48H Epidural analgesia | Usual care | Changes in creatinine | AKIN |
| Hansdottir 2006 | Superiority | 1 | Sweden | NR | 2002-2003 |  | 96H Epidural analgesia | Usual care | Length of hospital stay | 50% increased creatinine |
| Svircevic 2011 | Superiority | 2 | Netherlands | NA | 2004-2007 |  | In ICU Epidural analgesia | Usual care | survival free from myocardial infarction, pulmonary complications, renal failure, and stroke. | 50% increased creatinine |
| Zawar 2015 | Superiority | 1 | India | NA | 2011-2014 | Off pump Elderly (>70yo) | 72H Epidural analgesia | Usual care | Postoperative complications, total intensive care unit stay and hospital stay | increased of creatinine >2mg/dl |
| Hyperoxia | | | | | | | | | | |
| Abou-Arab 2019 | Superiority | 2 | France | Public | 2016-2018 | On-Pump | FiO2=1 during CPB | PaO2<150mmHg | POAF and/or ventricular fibrillation/tachycardia | KDIGO |
| McGuiness 2016 | Superiority | 2 | Australia, New-Zealand | Public | 2012-2014 | On-Pump | PaO2 75-90mmHg | Usual care | AKI | KDIGO |
| Onur 2020 | Superiority | 1 | Turkey | NR | 2019-2020 | On-Pump CABG | PaO2 100-180mmHg | PaO2 >180mmHg | Cognitive function | KDIGO |
| KDIGO care bundle | | | | | | | | | | |
| Meersch 2017 | Superiority | 1 | Germany | Mixed | 2014-2015 | [TIMP-2]·[IGFBP7] ≥ 0.3 (Nephrocheck® Test) 4 h after CPB. | « Bundle KDIGO » | Usual care | AKI | KDIGO |
| Montero 2017 | NA | 1 | Spain | NR | 2014-2016 |  | « Bundle KDIGO » | Usual care | Incidence of AKI | KDIGO |
| Zarbock 2021 | Superiority | 12 | Europe | Public | 2017-2019 | [TIMP-2]·[IGFBP7] ≥ 0.3 (Nephrocheck® Test) 4 h after CPB. | « Bundle KDIGO » | Usual care | Compliance to KDIGO bundle | KDIGO |
| High-target arterial pressure | | | | | | | | | | |
| Azau 2012 | Superiority | 1 | France | Public | 2008-2010 | CKD  or risk of AKI | MAP 75-85 mmHg | MAP 50-60 mmHg | Serum creatinine increased | 30% increased creatinine |
| Kandler 2019 | Superiority | 1 | Denmark | Public | 2011-2013 | Elderly (>70yo) | MAP>60mmHg | Usual care | Change in GFR after surgery | RIFLE |
| Vedel 2018 | Equivalence | 1 | Denmark | Public | 2014-2016 |  | MAP 70-80 mmHg | MAP 40-50mmHg | Volume of new ischemic lesions | 2-fold increased creatinine |
| Restrictive transfusion strategy | | | | | | | | | | |
| Fischer 2020 | Superiority | 2 | France | Public | 2017-2019 | On-pump | Hb>7.5g/dL and ScVO2>70% | Hb>9g/dL | Number of subjects receiving a transfusion | KDIGO |
| Hajjar 2010 | Non-inferiority | 1 | Brazil | NR | 2009-2010 |  | Ht>24% | Ht>30% | Composite of 30-day mortality and severe morbidity | RIFLE |
| Koch 2017 | Superiority | 2 | USA | Private | 2007-2014 |  | Ht>24% | Ht>28% | Composite of in hospital postoperative morbidity and mortality | NR |
| Mazer 2017 | Non-inferiority | 73 | 19 | Mixed | 2014-2017 | Euroscore>6 | Hb>7.5g/dL | Hb>9.5g/dL | Composite of death, MI, stroke, or new-onset renal failure with dialysis | KDIGO |
| Murphy 2015 | Superiority | 17 | UK | Public | 2009-2013 |  | Hb>7.5g/dL | Hb>9.5g/dL | Composite of a serious infection or an ischemic event | AKIN |
| Shehata 2012 | NR | 1 | Canada | NR | 2007-2010 | Care score 3-4 | Intraoperative: 7g/dL Postoperative: 7.5g/dL | Intraoperative: 9.5g/dL Postoperative: 10g/dL | Adherence to the transfusion strategies | RRT- or > 50% increased creatinine |
| Tight glycemic control | | | | | | | | | | |
| Asida 2013 | Superiority | 1 | Egypt | NR | 2010-2011 | On-pump Diabetic ASA 2/3 | BG 80-110 mg/dL | BG 110-180 mg/dL | Post-operative problems | creatinine>2mg/dl |
| Chan 2009 | Superiority | 1 | Brazil | Private | 2008-2009 | >21yo | BG 80-130 mg/dL | BG 160-200 mg/dL | Time of MV, length of stay in the ICU, infection, hypoglycemia, renal or neurological dysfunction, blood transfusion and length of stay in the hospital | Increased creatinine>50% |
| Desai 2012 | Both | 1 | US | No | 2007-2009 | CABG Diabetic or BG>150 | BG 90-120 mg/dL | BG 121-180 mg/dL | time to target glucose range, amount of insulin given, number of readings in target range, and number of patients with hypoglycemic events | NR |
| Ducan 2018 | Superiority | 2 | USA, Canada | Public | 2007-2015 | On-pump | BG 80-110 mg/dL | BG>150mg/dl | Composite: mortality 30day/mechanical circulatory support/infection/renal/neurologic morbidity | RRT |
| Ghandi 2007 | Superiority | 1 | USA | Mixed | 2004-2005 | On-pump | BG 80-100 mg/dL | BG<200mg/dl | Composite of death, sternal infections, prolonged ventilation, cardiac arrhythmias, stroke, and renal failure | NR |
| Kirdemir 2008 | Superiority | 2 | Turkey | NR | 2005-2007 | On-pump | BG 100-150 mg/dL | BG<200mg/dl | Supraventricular tachycardia | RRT |
| Mohod 2019 | NR | 1 | India | NR |  | CABG | BG 80-110 | BG<200mg/dl | incidence of hyperglycemia and hypoglycemia | NR |
| Santana-Santos 2019 | Superiority | 1 | Brazil | No | 2016-2016 | BG>200mg/dL within H6 post-operative | BG 90-110 mg/dL | BG140-180 mg/dL | AKI | KDIGO |
| Wahby 2016 | Superiority | 1 | Egypt | Public | 2013-2015 | On-pump CABG Diabetic | BG 110-149 mg/dL | BG 150-200 mg/dL | Mortality | Creatinine>2 mg/dl postoperative or more than 25% increased |
| Zadeh 2020 | Superiority | 1 | Iran | Public | 2017-2018 | On-pump CABG Diabetic | BG<120mg/dl | BG<200mg/dl | Mortality, sternal wound infection, cardiac arrhythmia, cerebrovascular attack and acute renal failure | 2-fold increased creatinine |

AKI: Acute kidney injury. AKIN: Acute Kidney Injury Network. ASA: American Society of Anesthesiology. BG: Blood Glucose. CABG: Coronary artery bypass graft. CPB: Cardiopulmonary bypass. CKD: Chronic kidney disease. CK MB: Creatine kinase MB. DO2: Oxygen delivery. GFR: Glomerular filtration rate. GI: Gastro-intestinal. Hb: Hemoglobin. Ht: Hematocrit. HsTnT: Hypersensitive troponin. IABP: Intra-aortic balloon pump. ICU: Intensive care unit. KDIGO: kidney disease improving global outcomes. KDOQI: Kidney Disease Outcomes Quality Initiative. LCOS: Low cardiac output syndrome. LVEF: Left ventricular ejection fraction. MACCE: Major adverse cardiac and cerebrovascular events. MECC: Minimally invasive extracorporeal circulation. MOF: Multiple organ failure. MI: myocardial infarction. MRI: Magnetic resonance imaging MV: Mechanical ventilation. NGAL: Neutrophil gelatinase-associated lipocalin. NR: Not reported. POAF: Post-operative atrial fibrillation. RCT: Randomized controlled trial. RIFLE: risk, injury, failure, loss of function and end- stage renal disease. RRT: Renal replacement therapy. SAP: Systolic arterial pressure.

| **Intervention** | **Trials with CKD as inclusion criteria** | **Trials with advanced CKD* as exclusion criteria** | **Number of trial reporting CKD** | **Patient with CKD in**  **Intervention group** | **Patients with CKD in control group** |
| --- | --- | --- | --- | --- | --- |
| GDP, N(%) | 0 (0) | 2 (100) | 1 (50) | 46 (33) | 40 (28) |
| RIPc, N(%) | 2 (6) | 17 (54) | 11 (35) | 322 (18) | 332 (19) |
| Pulsatile flow during CPB, N(%) | 0 (0) | 4 (40) | 3 (30) | 27 (10) | 27 (11) |
| MECC, N(%) | 0 (0) | 7 (50) | 3 (21) | 13 (6) | 11 (7) |
| Restrictive transfusion strategy, N(%) | 0 (0) | 2 (33) | 6 (100) | 1300 (15) | 1332 (15) |
| Epidural analgesia, N(%) | 0 (0) | 0 (0) | 2 (50) | 3 (2) | 6 (5) |
| Tight glycemic control, N(%) | 0 (0) | 4 (40) | 5 (50) | 19 (6) | 20 (6) |
| KDIGO care, N(%) | 0 (0) | 3 (100) | 3 (100) | 40 (15) | 41 (13) |
| High-Target arterial pressure, N(%) | 1 (33) | 1 (33) | 1 (33) | 84 (57) | 73 (50) |
| Hyperoxia during CPB, N(%) | 0 (0) | 1 (33) | 1 (33) | 20 (12) | 10 (6) |

**eTable3. Characteristics of chronic kidney disease population for each intervention**

* Glomerular filtration rate < 30ml/min/m^2^ or patients on dialysis

GDP: goal-directed perfusion; RIPc, remote ischemic preconditioning; MECC, minimally invasive extracorporeal circulation; CPB, cardiopulmonary bypass; KDIGO, Kidney Disease Improving Global Outcomes; CKD : Chronic kidney disease. NR: Not reported.

**eFigure 1. Risk of bias of randomized controlled trials assessing a non-pharmacological intervention to prevent cardiac surgery associated - acute kidney injury**

**eFigure 2a. Definition of cardiac surgery associated acute kidney injury in all included RCTs according to each intervention**

AKIN: Acute Kidney Injury Network. RIFLE: risk, injury, failure, loss of function and end- stage renal disease. RRT: Renal replacement therapy. RCTs: Randomized controlled trials. GDP: Goal directed perfusion. RIPc: Remote ischemic preconditioning. CPB: Cardiopulmonary bypass. MECC: Minimally invasive extracorporeal circulation. KDIGO: kidney disease improving global outcomes. IQR: Interquartile Range

**eFigure 2b. Acute kidney injury definition according to the year of publication of the trials**

AKIN: Acute Kidney Injury Network.RCTs. RIFLE: risk, injury, failure, loss of function and end- stage renal disease. KDIGO: kidney disease improving global outcomes. RCTs: Randomized controlled trials.

**eFigure 3. Meta-analysis of the effect of goal directed perfusion (GDP) on cardiac surgery associated acute kidney injury**.

Risk of bias: A: Random sequence generation, B: Allocation concealment, C: Blinding of participants and personnel, D: Incomplete outcome data, E: Selective reporting, F: Overall bias.

**eFigure 4. Subgroup analysis of the effect of Remote ischemic preconditioning (RIPc) on cardiac surgery associated acute kidney injury according the Risk of bias**


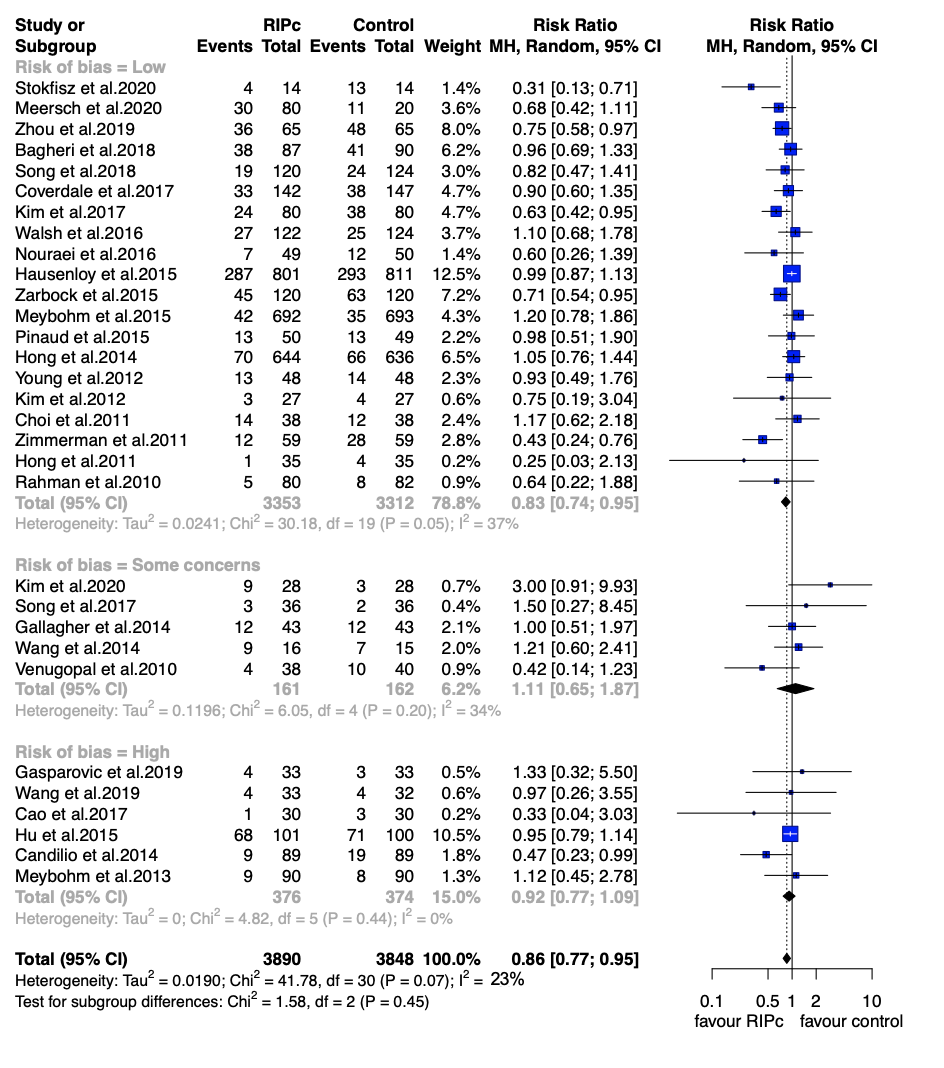


**
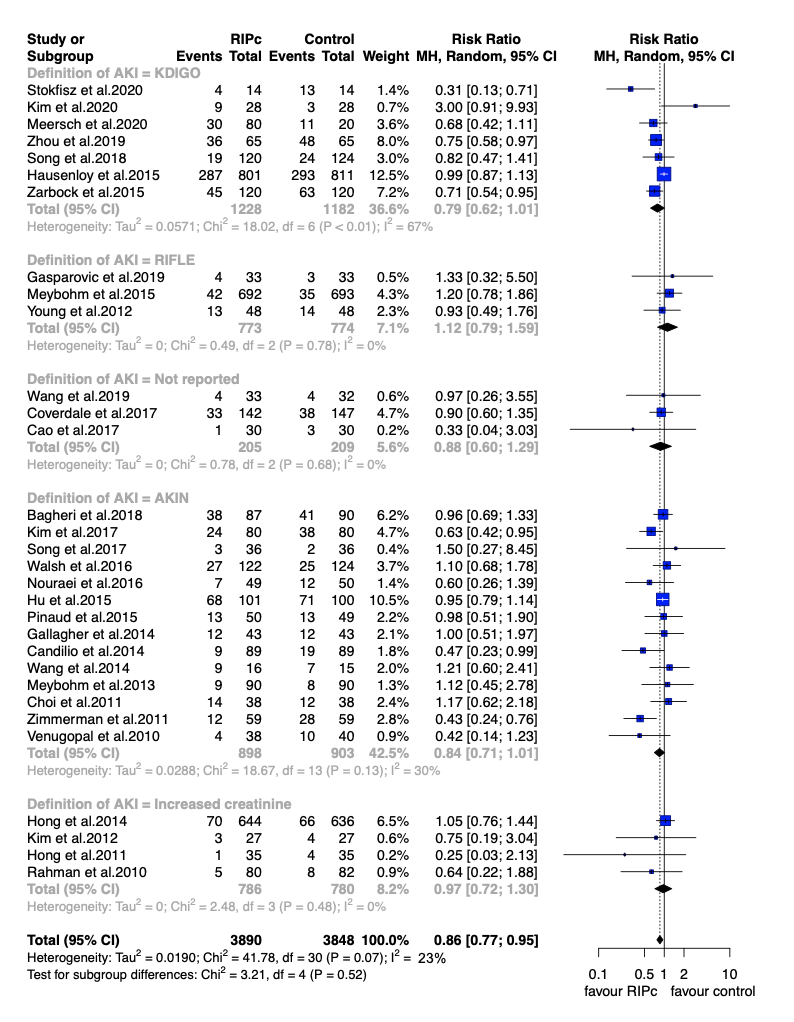
eFigure 5. Subgroup analysis of the effect of Remote ischemic preconditioning (RIPc) on cardiac surgery associated acute kidney injury according the definition of acute kidney injury (AKI)**

AKIN: Acute Kidney Injury Network.

**eFigure 6. Subgroup analysis of the effect of Remote ischemic preconditioning (RIPc) on cardiac surgery associated acute kidney injury according the type of surgery.**

CABG: coronary artery bypass graft

**eFigure 7. Funnel plot for random effects meta-analysis of cardiac surgery associated acute kidney injury outcomes in trials of Remote ischemic preconditioning**

**eFigure 8. Meta-analysis of the effect of pulsatile flow on cardiac surgery associated acute kidney injury.**

Risk of bias: A: Random sequence generation, B: Allocation concealment, C: Blinding of participants and personnel, D: Incomplete outcome data, E: Selective reporting, F: Overall bias

**eFigure 9. Subgroup analysis of the effect of pulsatile flow on cardiac surgery associated acute kidney injury according the modality of pulsatility.**

**
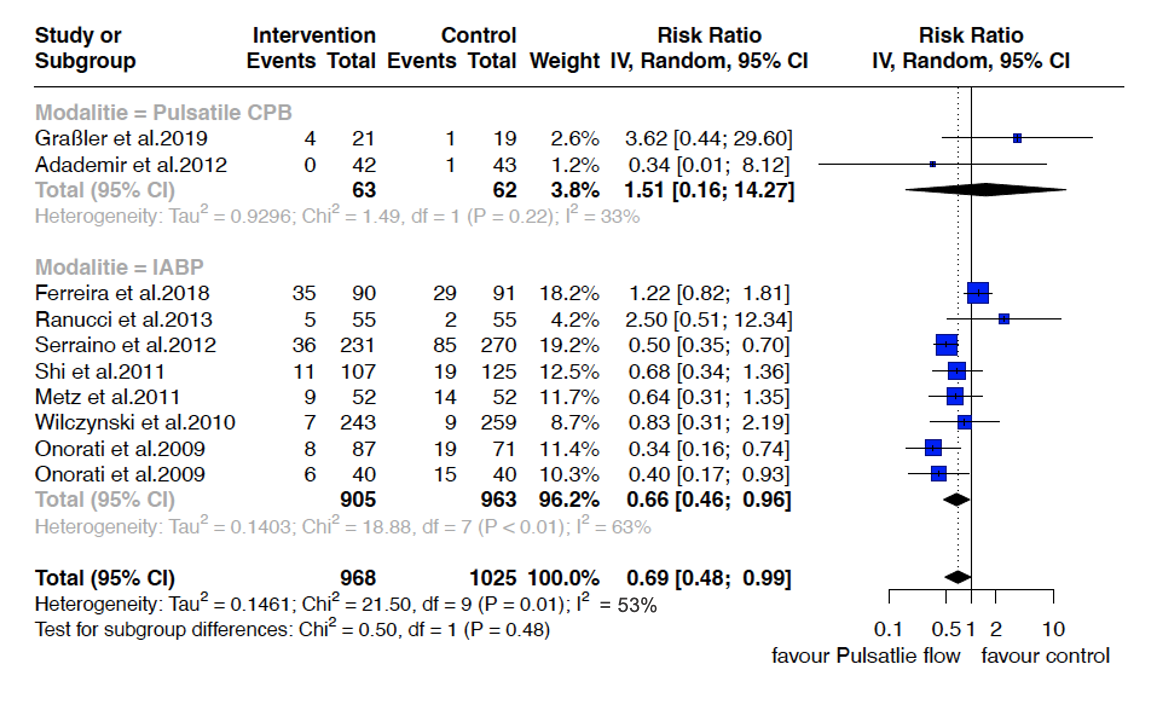
**

CPB: cardiopulmonary bypass.IABP: Intra-aortic balloon pump

**eFigure 10. Subgroup analysis of the effect of pulsatile flow on cardiac surgery associated acute kidney injury according the definition of acute kidney injury (AKI)**


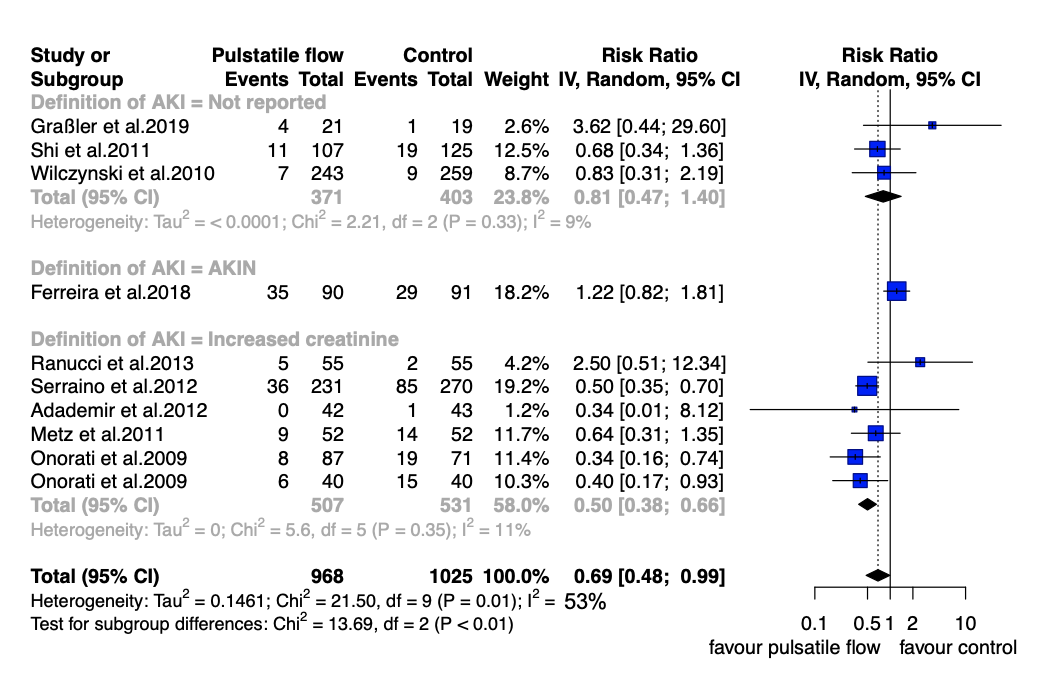


AKIN: Acute Kidney Injury Network.

**eFigure 11. Subgroup analysis of the effect of pulsatile flow on cardiac surgery associated acute kidney injury according the risk of bias**


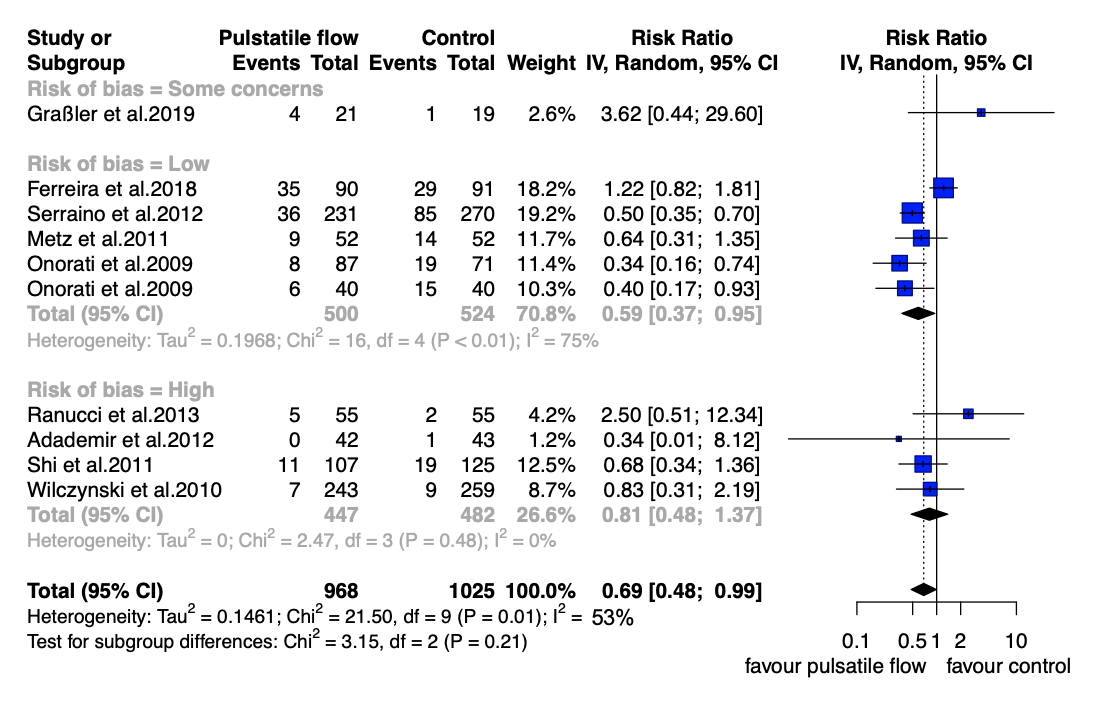


****eFigure 12. Funnel plot for random effects meta-analysis of CSA-AKI outcomes in RCTs of Pulsatile flow during CPB**

**eFigure 13. Meta-analysis of the effect of Minimally invasive extracorporeal circulation (MECC) on cardiac surgery associated acute kidney injury.**

Risk of bias: A: Random sequence generation, B: Allocation concealment, C: Blinding of participants and personnel, D: Incomplete outcome data, E: Selective reporting, F: Overall bias

**eFigure 14. Subgroup analysis of the effect of Minimally invasive extracorporeal circulation (MECC) on cardiac surgery associated acute kidney injury according the risk of bias**


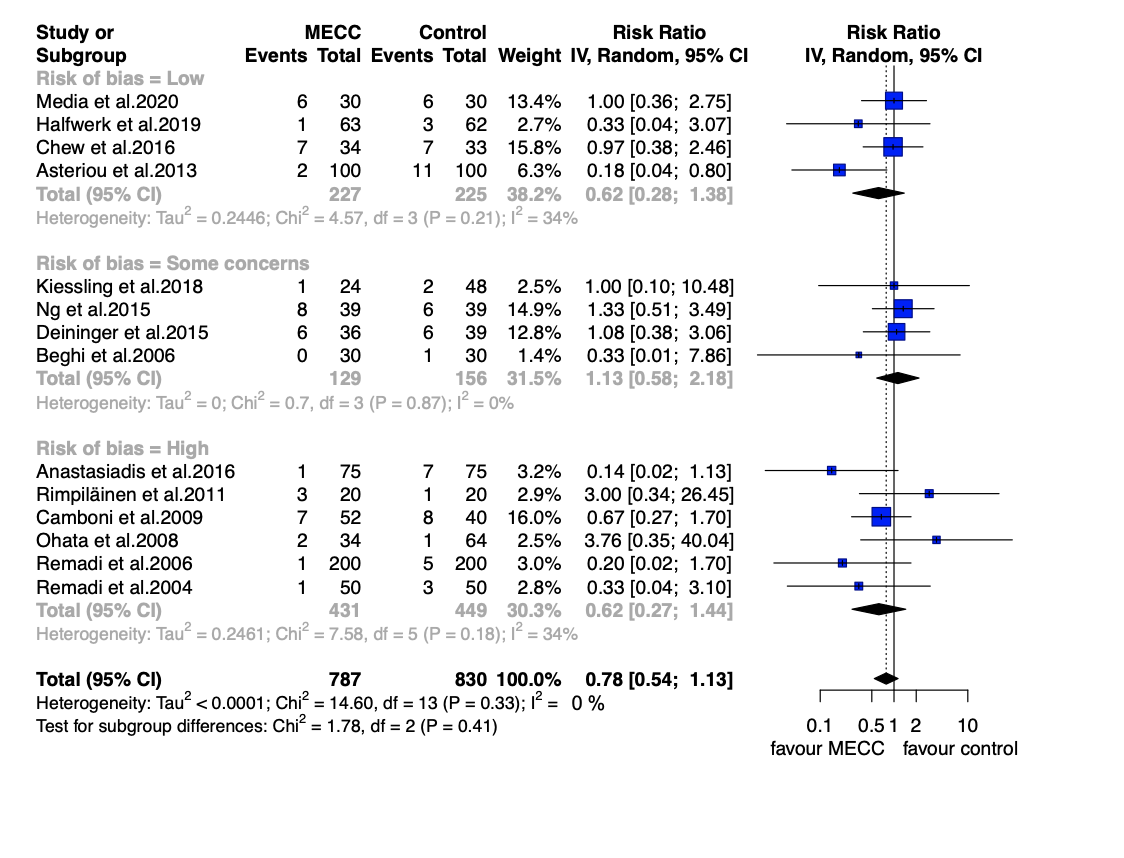


**eFigure 15. Subgroup analysis of the effect of Minimally invasive extracorporeal circulation (MECC) on cardiac surgery associated acute kidney injury according the definition of acute kidney injury (AKI)**


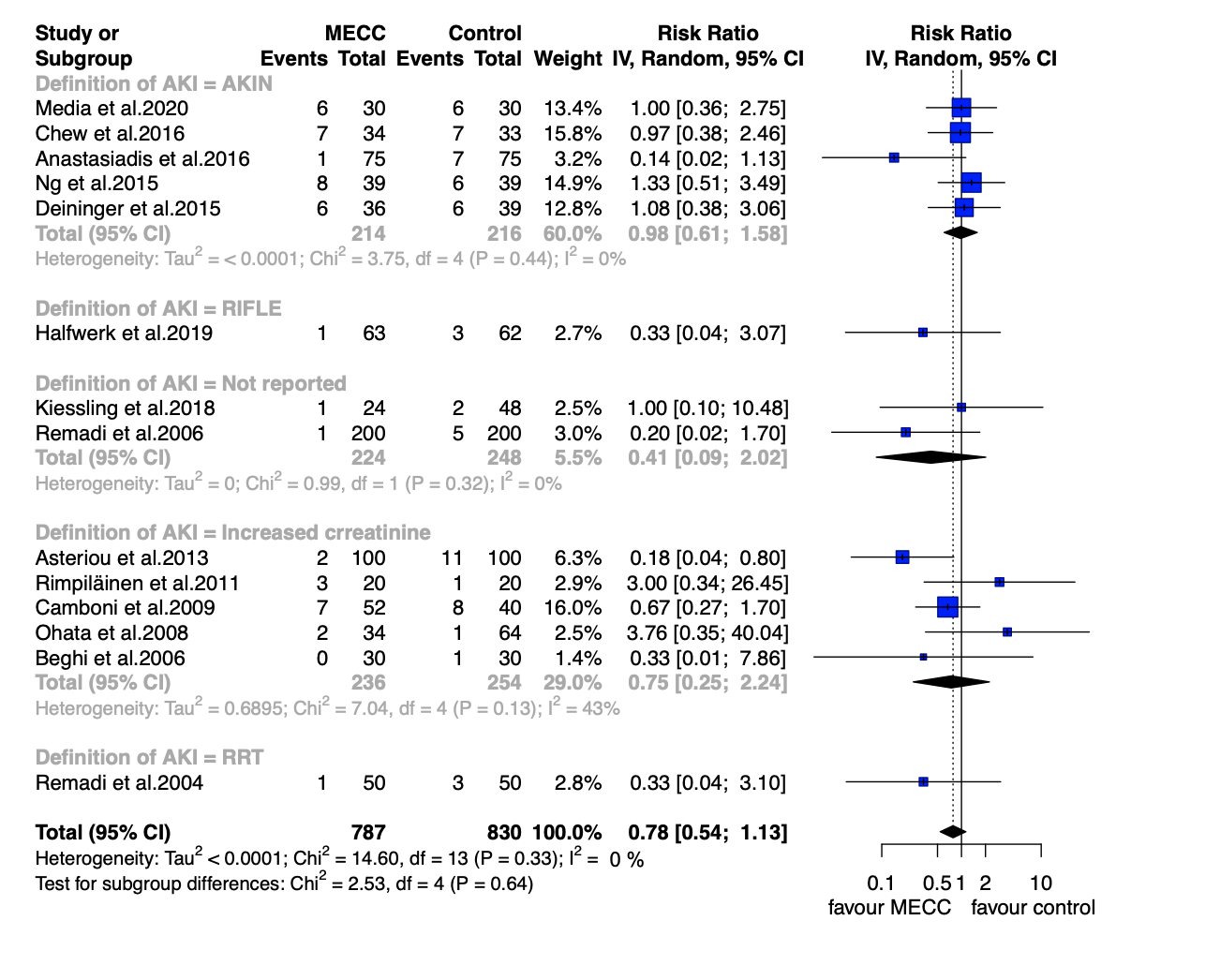


AKIN: Acute Kidney Injury Network. RIFLE: risk, injury, failure, loss of function and end- stage renal disease. RRT: Renal Replacement Therapy

**eFigure 16. Subgroup analysis of the effect of Minimally invasive extracorporeal circulation (MECC) on cardiac surgery associated acute kidney injury according the type of surgery**

CABG: coronary artery bypass graft

**eFigure 17. Funnel plot for random effects meta-analysis of cardiac surgery associated acute kidney injury outcomes in trials of Minimally invasive extracorporeal circulation (MECC)**

******

**eFigure 18. Meta-analysis of the effect of restrictive transfusion strategy on cardiac surgery associated acute kidney injury.**

Risk of bias: A: Random sequence generation, B: Allocation concealment, C: Blinding of participants and personnel, D: Incomplete outcome data, E: Selective reporting, F: Overall bias

**eFigure 19. Subgroup analysis of the effect of restrictive transfusion strategy on cardiac surgery associated acute kidney injury according the risk of bias**

**
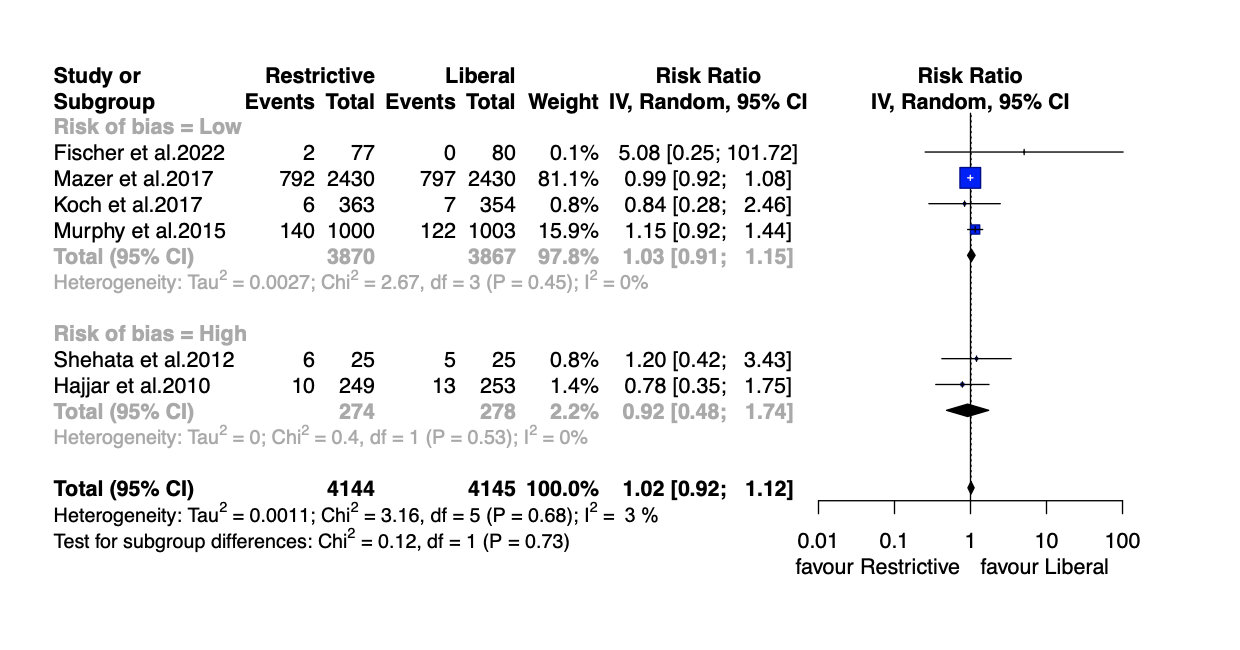
**

**eFigure 20. Subgroup analysis of the effect of restrictive transfusion strategy on cardiac surgery associated acute kidney injury according the definition of acute kidney injury (AKI)**


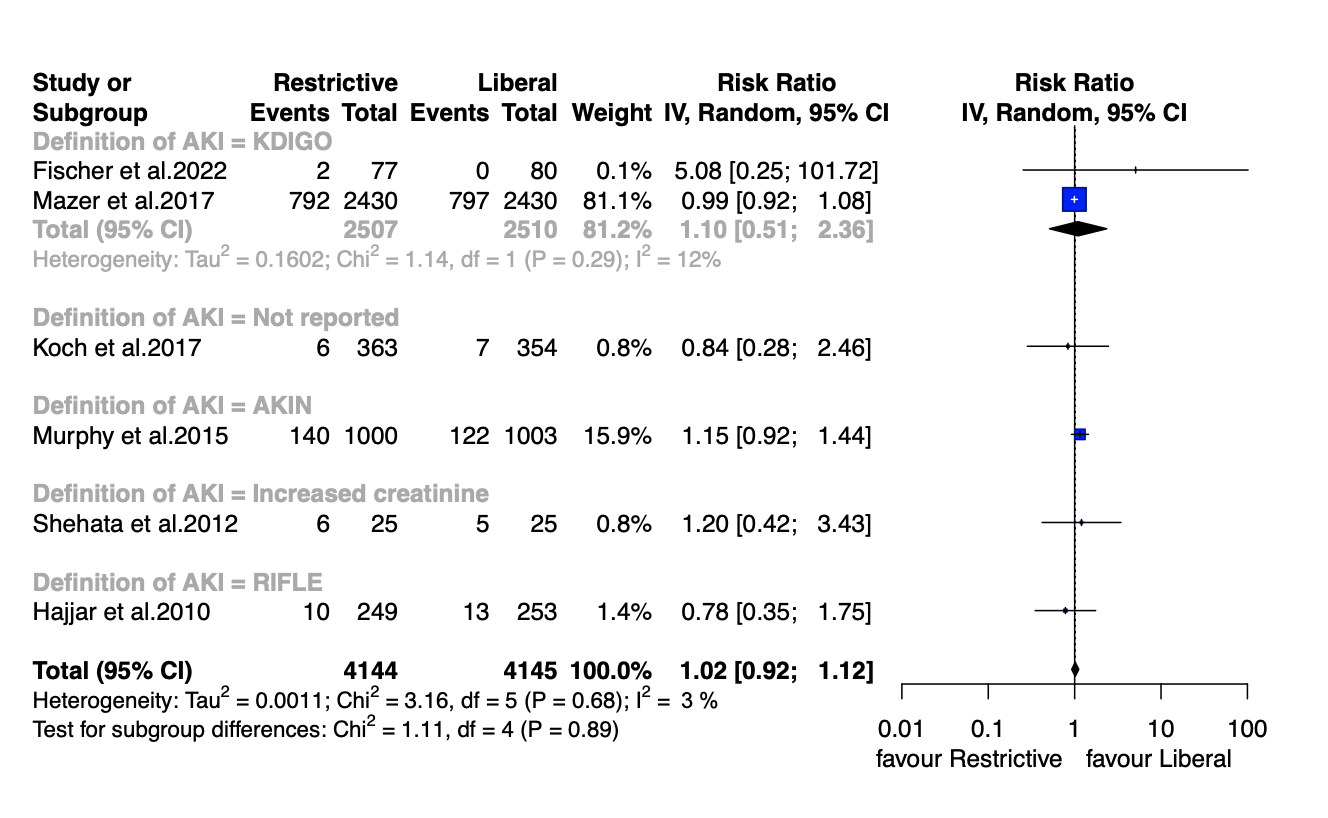


AKIN: Acute Kidney Injury Network.RIFLE: risk, injury, failure, loss of function and end- stage renal disease. KDIGO: kidney disease improving global outcomes.

**eFigure 21. Meta-analysis of the effect of tight glycemic (BG) control on cardiac surgery associated acute kidney injury**

Risk of bias: A: Random sequence generation, B: Allocation concealment, C: Blinding of participants and personnel, D: Incomplete outcome data, E: Selective reporting, F: Overall bias

**eFigure 22. Subgroup analysis of the effect of tight glycemic (BG) control on cardiac** **surgery associated acute kidney injury according the risk of bias**

**
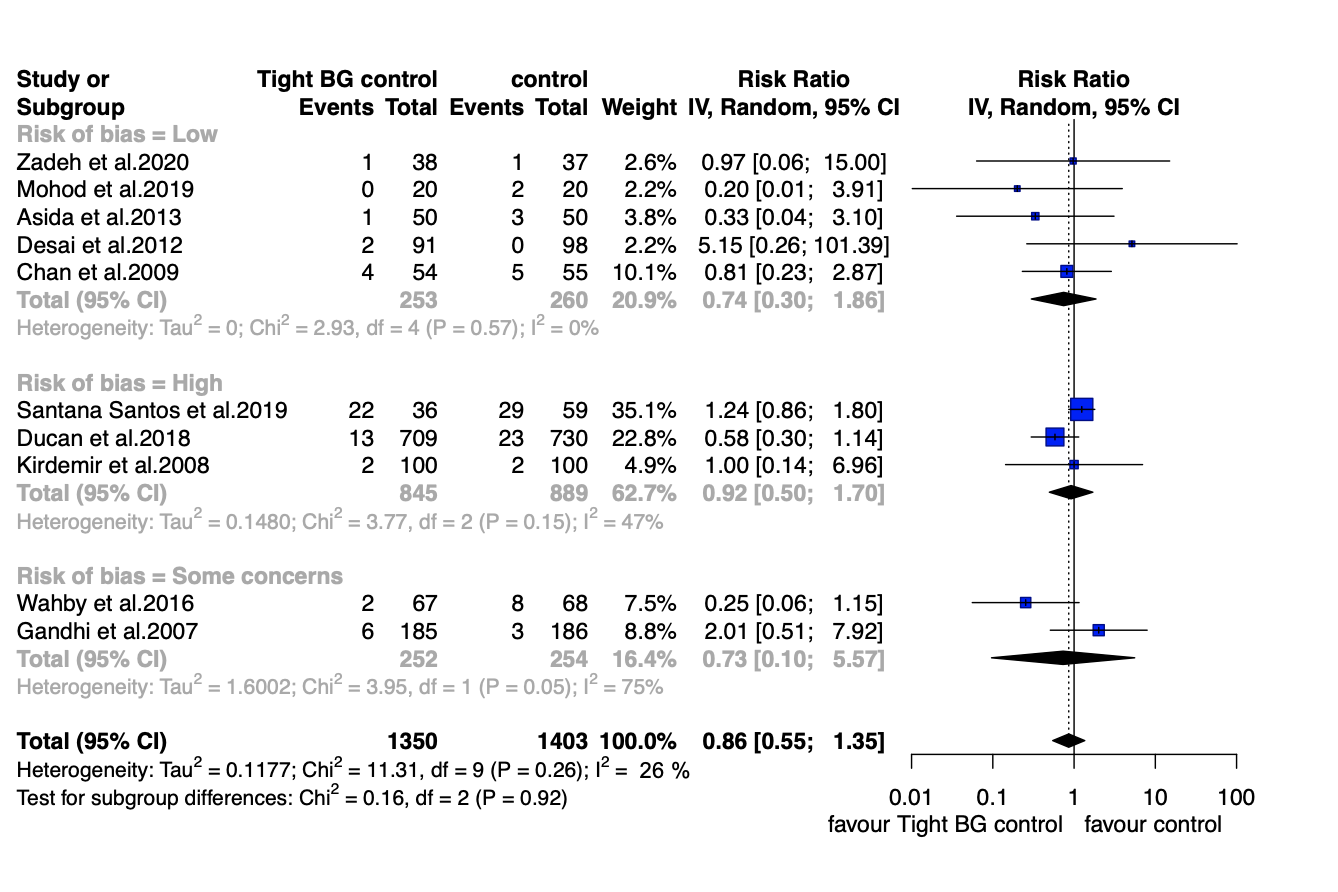
**

**eFigure 23. Subgroup analysis of the effect of tight glycemic (BG) control on cardiac surgery associated acute kidney injury according the definition of acute kidney injury (AKI)**


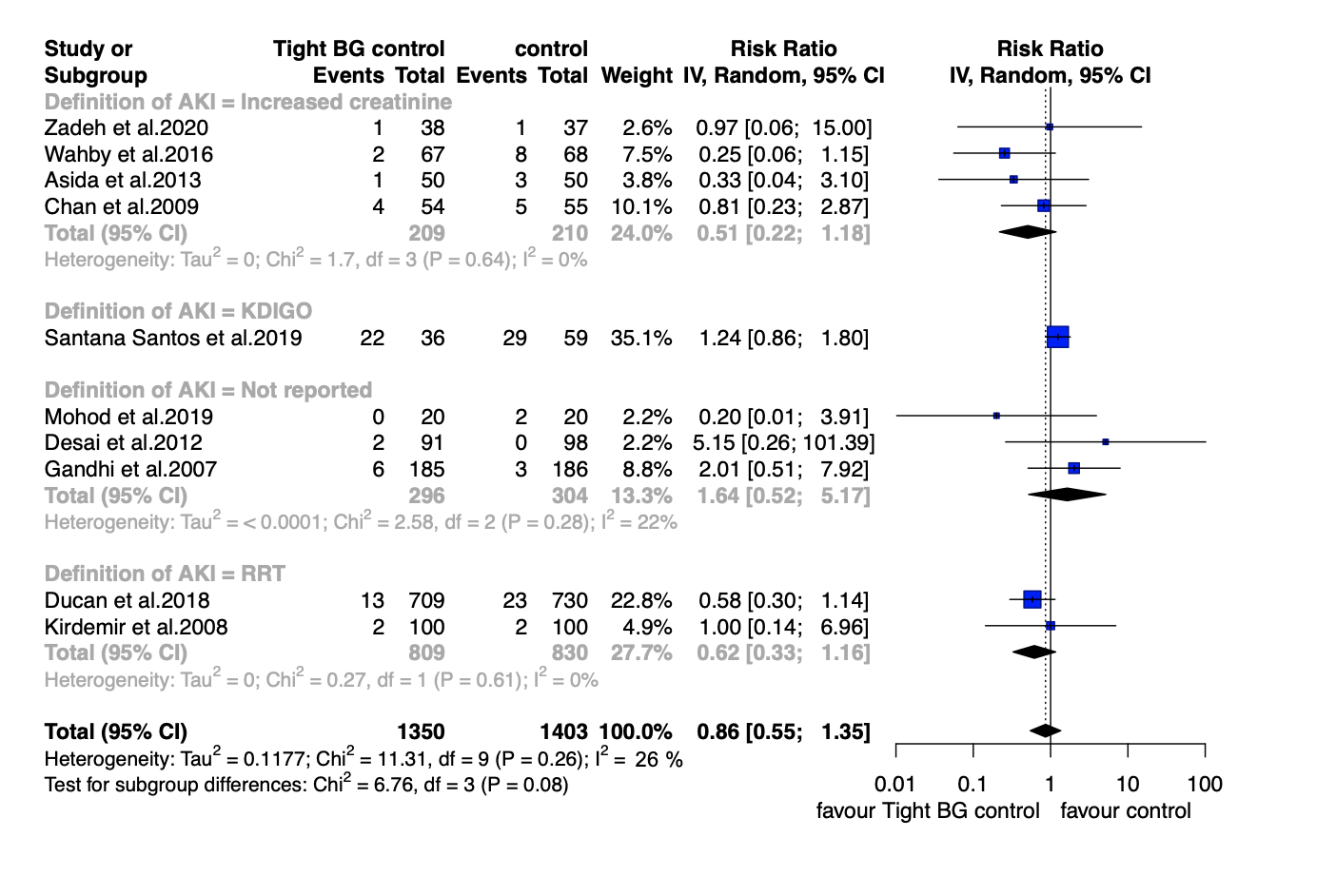


KDIGO: kidney disease improving global outcomes. RRT: Renal Replacement Therapy

**eFigure 24. Funnel plot of tight glycemic control on cardiac surgery associated acute kidney injury**

**eFigure 25. Meta-analysis of the effect of Epidural analgesia on cardiac surgery associated acute kidney injury**.

Risk of bias: A: Random sequence generation, B: Allocation concealment, C: Blinding of participants and personnel, D: Incomplete outcome data, E: Selective reporting, F: Overall bias

**eFigure 26. Subgroup analysis of the effect of Epidural analgesia on cardiac surgery associated acute kidney injury according the risk of bias**


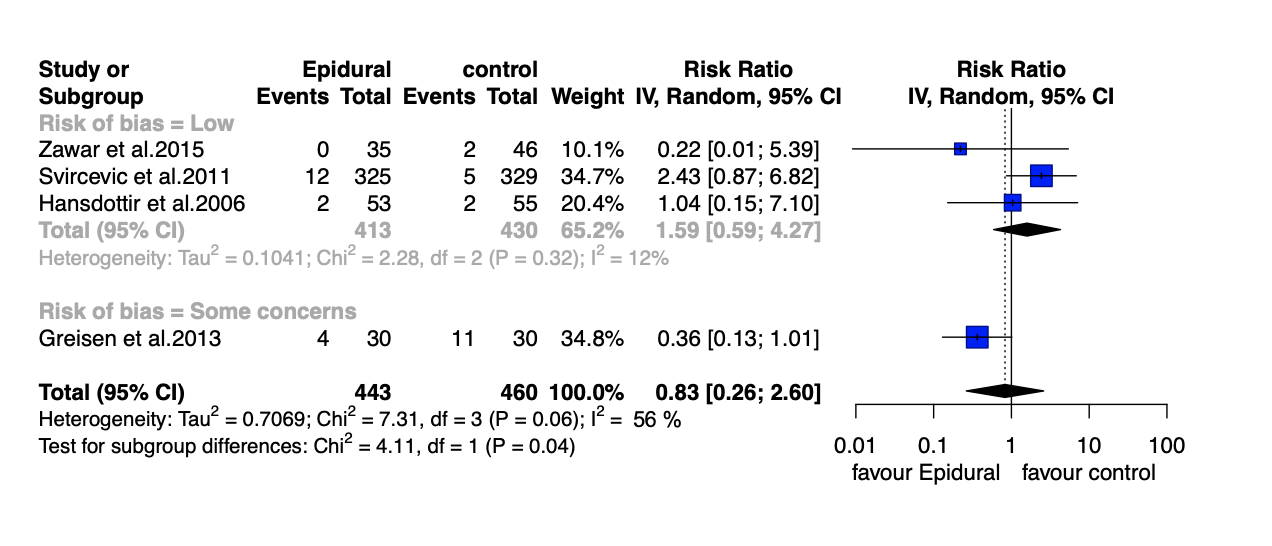


**eFigure 27. Subgroup analysis of the effect of Epidural analgesia on cardiac surgery associated acute kidney injury according the definition of acute kidney injury (AKI)**


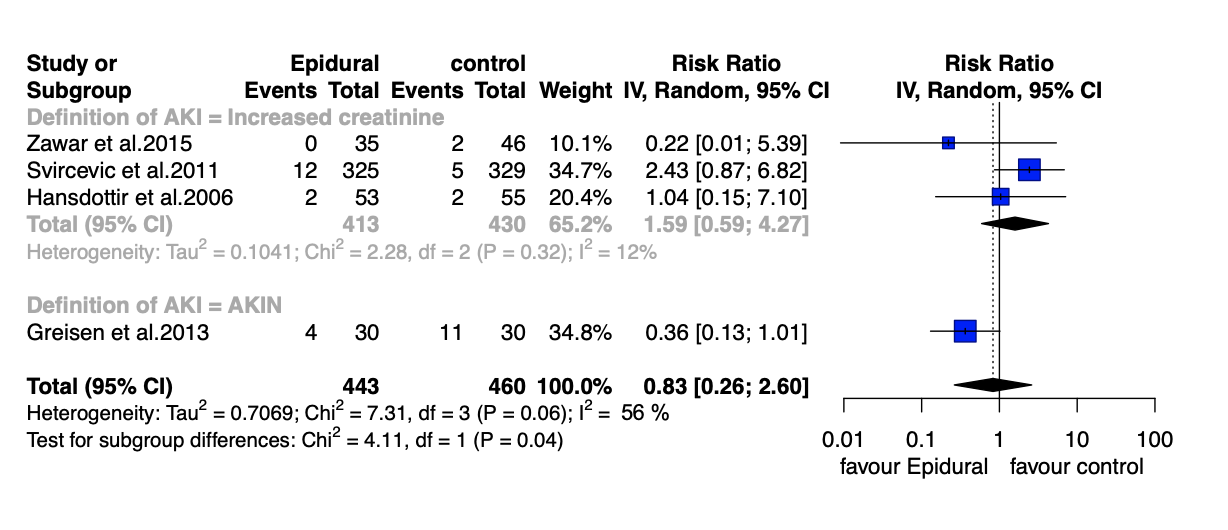


AKIN: Acute Kidney Injury Network.

**eFigure 28. Meta-analysis of the effect of KDIGO bundle of care on cardiac surgery associated acute kidney injury**

Risk of bias: A: Random sequence generation, B: Allocation concealment, C: Blinding of participants and personnel, D: Incomplete outcome data, E: Selective reporting, F: Overall bias. KDIGO: kidney disease improving global outcomes

**eFigure 29. Subgroup analysis of the effect of KDIGO care on cardiac surgery associated acute kidney injury according the risk of bias**


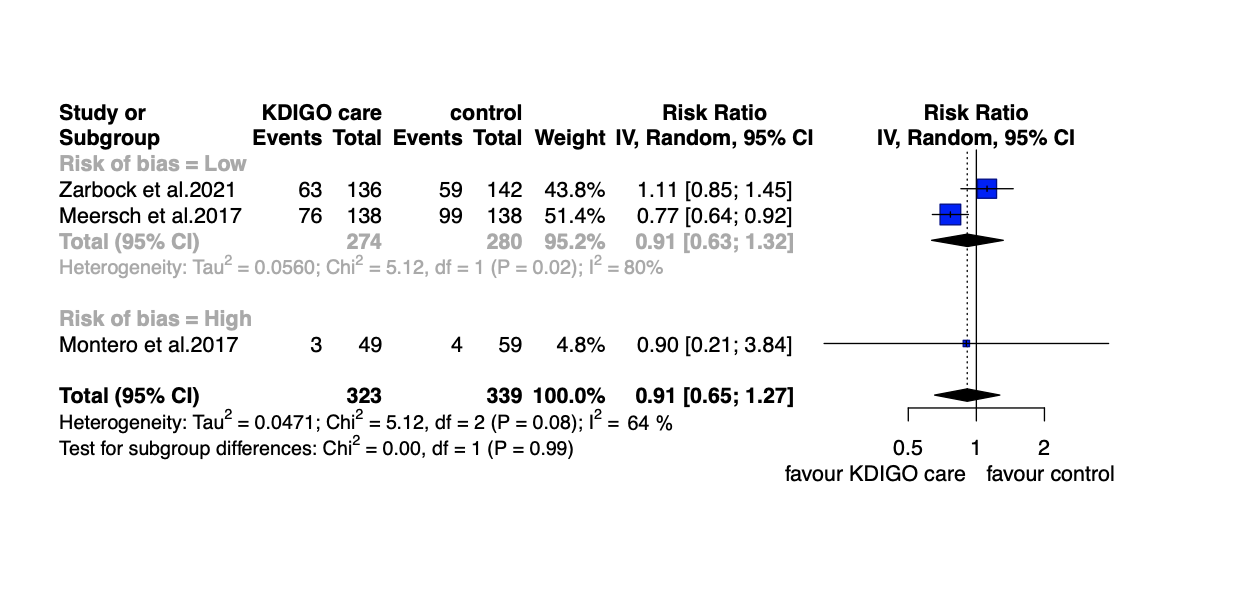


KDIGO: kidney disease improving global outcomes

**eFigure 30. Meta-analysis of the effect of high-target arterial pressure (MAP) target on cardiac surgery associated acute kidney injury.**

Risk of bias: A: Random sequence generation, B: Allocation concealment, C: Blinding of participants and personnel, D: Incomplete outcome data, E: Selective reporting, F: Overall bias

**eFigure 31. Subgroup analysis of the effect of high-target arterial pressure (MAP) target on cardiac surgery associated acute kidney injury according the definition of acute kidney injury (AKI)**


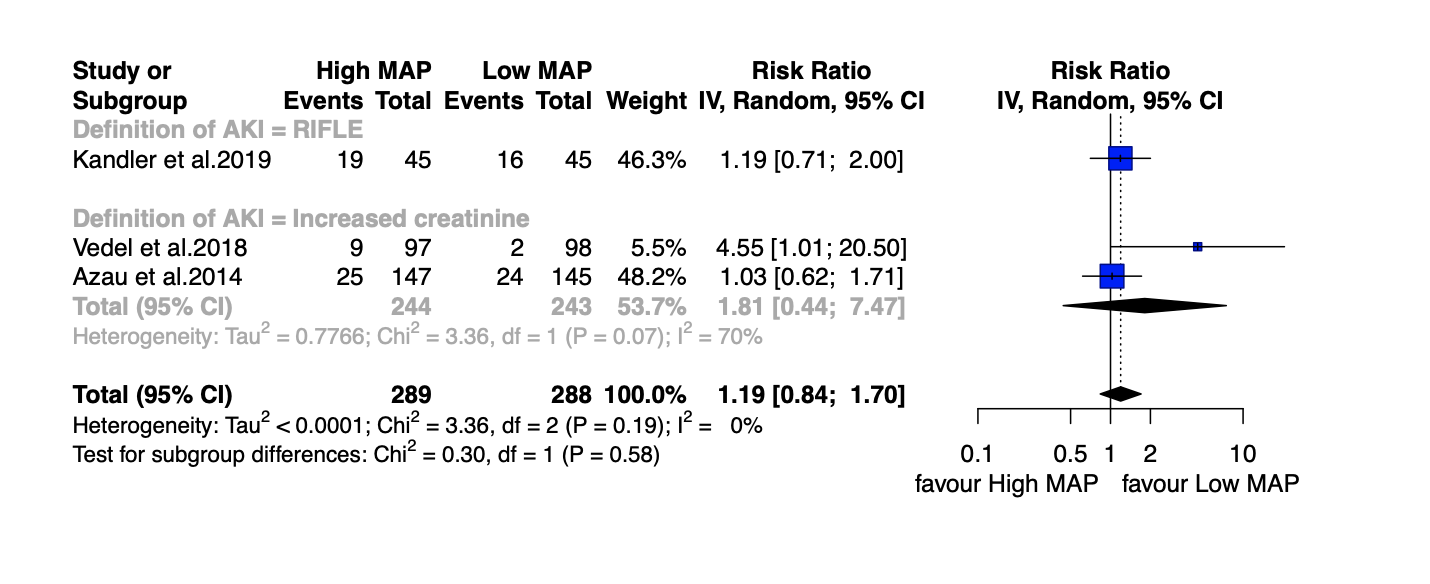


RIFLE: risk, injury, failure, loss of function and end- stage renal disease.

**eFigure 32. Meta-analysis of the effect of hyperoxia on cardiac surgery associated acute kidney injury.**

Risk of bias: A: Random sequence generation, B: Allocation concealment, C: Blinding of participants and personnel, D: Incomplete outcome data, E: Selective reporting, F: Overall bias.

**eFigure 33. Subgroup analysis of the effect of hyperoxia on cardiac surgery associated acute kidney injury according the risk of bias**

**eFigure 34. Forest plot of non-pharmacological interventions for efficacy in reducing risk of mortality. Interventions were tested with standard medical care as control.**

CPB=cardiopulmonary bypass; KDIGO=kidney disease improving global outcomes

**eFigure 35. Forest plot of non-pharmacological interventions for efficacy in reducing risk of RRT. Interventions were tested with standard medical care as control.**

CPB=cardiopulmonary bypass; KDIGO=kidney disease improving global outcomes

**eFigure 36 Forest plot of non-pharmacological interventions for efficacy in reducing lenght of hospital stay. Interventions were tested with standard medical care as control.**

CPB=cardiopulmonary bypass; KDIGO=kidney disease improving global outcomes

**eFigure 37 Forest plot of non-pharmacological interventions for efficacy in reducing length of Intensive care unit stay. Interventions were tested with standard medical care as control.**

CPB=cardiopulmonary bypass; KDIGO=kidney disease improving global outcomes

**eFigure 38. Forest plot of non-pharmacological interventions for efficacy in reducing risk of post-operative stroke. Interventions were tested with standard medical care as control.**

CPB=cardiopulmonary bypass; KDIGO=kidney disease improving global outcomes

**eFigure 39. Forest plot of non-pharmacological interventions for efficacy in reducing risk of post-operative myocardial infarction. Interventions were tested with standard medical care as control.**

CPB=cardiopulmonary bypass

**eFigure 40. Forest plot of non-pharmacological interventions for efficacy in reducing risk of post-operative atrial fibrillation. Interventions were tested with standard medical care as control.**

CPB=cardiopulmonary bypass; KDIGO=kidney disease improving global outcomes


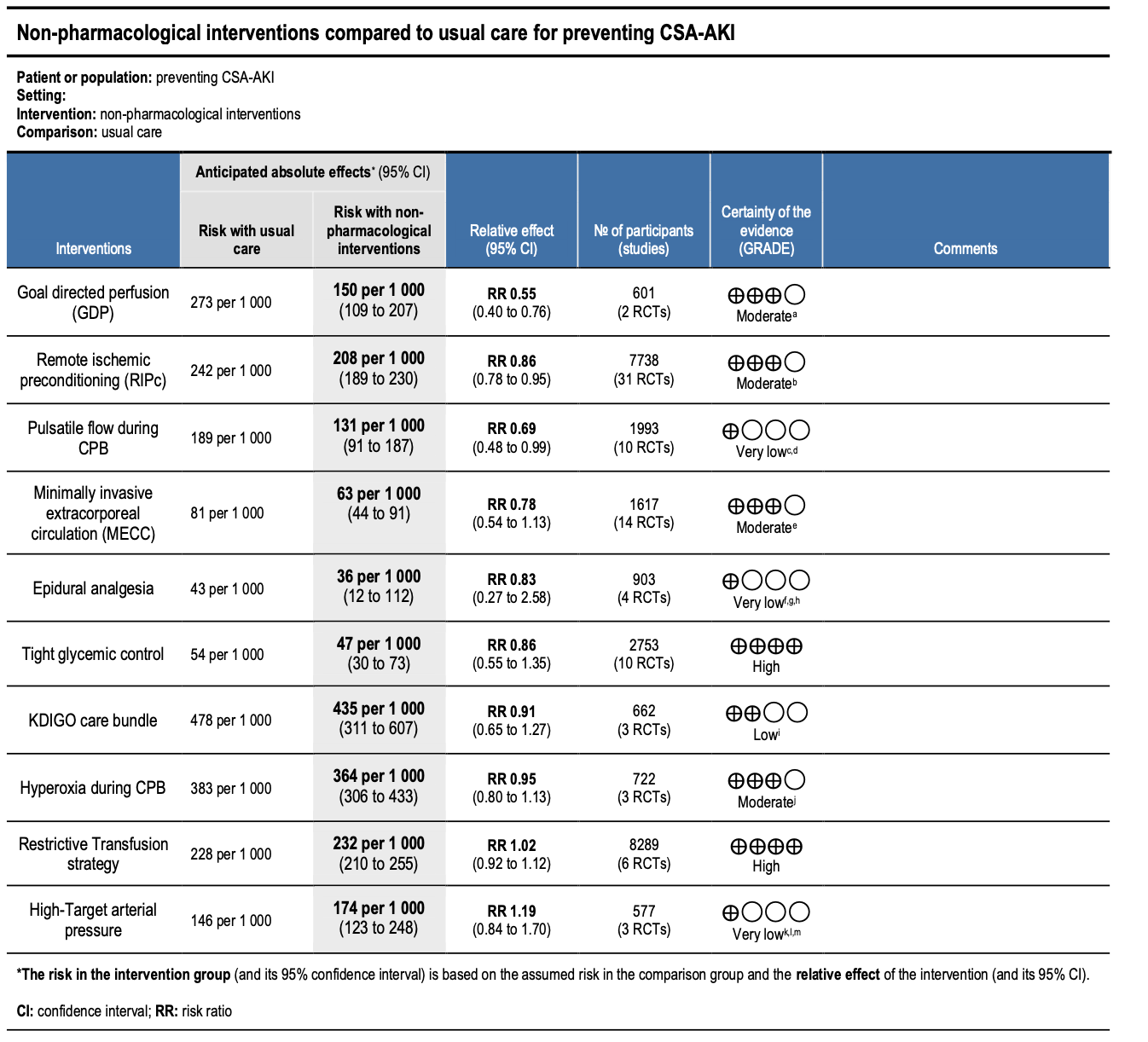
**eTable 4. Summary of quality of evidence for the effect of each intervention on CSA-AKI according to the Grading of Recommendations Assessment, Development, and Evaluation (GRADE).**

a. Downgraded because evaluated by only 2 RCTs

b. Downgraded because asymmetric funnel plot

c. Downgraded because half of the trials had at least some concerns risk of bias

d. Downgraded because we detected heterogeneity

e. Downgraded because most of the trials had some concerns risk of bias

f. Downgraded because all trials had risk of bias

g. Downgraded because we detected heterogeneity

h. Downgraded because of large confidence interval

i. Downgraded because we detected heterogeneity

j. Downgraded because evaluated by only 3 RCTs

k. Downgraded because only one trial was at low risk of bias

l. Downgraded because we detected heterogeneity

m. Downgraded because of large confidence interval

RR, risk ratio
